# Effective sequence similarity detection with strobemers

**DOI:** 10.1101/gr.275648.121

**Published:** 2021-11

**Authors:** Kristoffer Sahlin

**Affiliations:** Department of Mathematics, Science for Life Laboratory, Stockholm University, 10691 Stockholm, Sweden

## Abstract

*k*-mer-based methods are widely used in bioinformatics for various types of sequence comparisons. However, a single mutation will mutate *k* consecutive *k*-mers and make most *k*-mer-based applications for sequence comparison sensitive to variable mutation rates. Many techniques have been studied to overcome this sensitivity, for example, spaced *k*-mers and *k*-mer permutation techniques, but these techniques do not handle indels well. For indels, pairs or groups of small *k*-mers are commonly used, but these methods first produce *k*-mer matches, and only in a second step, a pairing or grouping of *k*-mers is performed. Such techniques produce many redundant *k*-mer matches owing to the size of *k*. Here, we propose *strobemers* as an alternative to *k*-mers for sequence comparison. Intuitively, strobemers consist of two or more linked shorter *k*-mers, where the combination of linked *k*-mers is decided by a hash function. We use simulated data to show that strobemers provide more evenly distributed sequence matches and are less sensitive to different mutation rates than *k*-mers and spaced *k*-mers. Strobemers also produce higher match coverage across sequences. We further implement a proof-of-concept sequence-matching tool StrobeMap and use synthetic and biological Oxford Nanopore sequencing data to show the utility of using strobemers for sequence comparison in different contexts such as sequence clustering and alignment scenarios.

The dramatic increase in sequencing data generated over the past two decades has prompted a significant focus on developing computational methods for sequence comparison. A popular sequence comparison paradigm is *k*-mer-based analysis, in which *k*-mers are substrings of length *k* of, for example, genomic, transcriptomic, or protein sequences. *k*-mer-based methods have been applied for sequence comparison for error correction (Salmela et al. 2016), genome assembly (Pevzner 1989; Chikhi and Medvedev 2014), metagenomic (Wood and Salzberg 2014) and chromosome (Rangavittal et al. 2019) sequence classification, sequence clustering (Steinegger and Söding 2018), database searches (Solomon and Kingsford 2016; Harris and Medvedev 2020), structural variation detection (Abo et al. 2015; Standage et al. 2019; Khorsand and Hormozdiari 2021), transcriptome analysis (Patro et al. 2014; Bray et al. 2016), DNA barcoding of species (Chor et al. 2009), estimation of genome size (Hozza et al. 2015), identification of biomarkers (Wang et al. 2018), and many other applications. Because of the widespread use of *k*-mers, many data structures and techniques for efficiently storing and querying *k*-mers have been proposed (for a review, see Marchet et al. 2021).

Although *k*-mers have proven to be practical in several sequence comparison problems, they are sensitive to mutations. A mutation will mutate *k* consecutive *k*-mers across a string. As the mutation rate increases, the number of matching *k*-mers between two sequences quickly reduces. In work by Blanca et al. (2021), the distribution of mutated *k*-mers was studied in detail. The investigators provided closed-form expressions for the mean and variance estimates on the number of mutated *k*-mers under a random mutation model. Although the number of *k*-mer matches between sequences is of interest, it is often more informative to know how they are distributed across the matching region. *k*-mer matches, because of their consecutive nature, cluster tightly in shared sequence regions, whereas matches may be absent in regions with higher mutation rates. A spaced *k*-mer (or spaced seed) is a *k*-mer replacement method that have been studied in several sequence comparison contexts to overcome the *k*-mers’ sensitivity to mutations (Ma et al. 2002; Burkhardt and Kärkkäinen 2003; Břinda et al. 2015; Wood et al. 2019). Spaced *k*-mers can produce matches over substitutions and produce less positionally correlated matches compared with *k*-mers. In fact, *k*-mers are in some conditions the worst seed pattern for the problem of similarity search (Keich et al. 2004). An approach that generalizes a spaced *k*-mer is a vector seed (Brejová et al. 2005). The vector seed approach introduces seeds with weighted positions and a seed distance threshold. The seeds between the reference and the query match if the weighted seed distance is lower than the threshold. In this context, a spaced *k*-mer can be seen as a seed in which the positions have a weight of zero or one. Vector seeds, similarly to spaced *k*-mers, are limited to handling only substitutions. In addition, matches are computationally more expensive to compute as a simple membership lookup of the seed pattern is not possible.

Another innovative idea has been to permute the letters in a string before comparison (Charikar 2002; Lederman 2013). The main idea is to permute the letters in regions of fixed size in a string using several different permutations. Then, when comparing two strings in the regions under these permutations, at least one permutation will, with statistical certainty, have pushed any substitution(s) toward the end of the region. This allows for a constant-time query of the prefix of the region in the permuted strings. With more permutations, it is more likely to find an exact prefix match. However, both spaced *k*-mers and permutation techniques are only practical for substitutions. An insertion or deletion (*indel*) will shift the sequence and, similarly to *k*-mers, result in a long stretch of dissimilar *k*-mers. For certain applications, such as genome assembly, selecting several sizes of *k* for inference has also been shown to help sequence comparison (Bankevich et al. 2012), but it significantly increases runtime and complexity of analysis. There are also methods to collapse repetitive regions before *k*-mer-based comparison (Liu et al. 2016), which reduces the processing time of repetitive hits. However, such techniques are usually used for reference-based analysis and do not apply to general sequence comparison problems. A notable approach that is related to the permutation approaches is covering template families (Giladi et al. 2010). In this work, the investigators propose a construction algorithm to produce a set of gapped seeds (templates) such that at least one of the seeds is guaranteed to match over both substitutions and indels, given some upper bound on the error rate. To achieve this guarantee, multiple seeds need to be extracted and compared between the query and the reference, which results in a high computational query cost and high memory usage when storing an index with the templates for the reference. Construction of multiple spaced seed patterns has also been presented to allow for matches up to one gap (Burkhardt and Kärkkäinen 2002) or up to some level of mismatches (Pevzner and Waterman 1995; Kucherov et al. 2005).

As third-generation sequencing techniques appeared with sequencing errors, mostly consisting of insertions and deletions, many of the previously developed sequence comparison techniques for short-read data became unsuitable. For third-generation sequencing data, MinHash (Broder 1997) and minimizers (Schleimer et al. 2003; Roberts et al. 2004) proved to be useful *k*-mer subsampling methods for such sequence comparison as minimizers can be preserved in a window affected by an indel. In addition, they also reduce the size of the index by subsampling the data. This has made MinHash and minimizers a popular technique for subsampling *k*-mers used for sequence comparison in a range of applications such as metagenome distance estimation (Ondov et al. 2016) and alignment (Li 2016; Jain et al. 2018, 2020a), clustering (Sahlin and Medvedev 2020) error correction (Sahlin and Medvedev 2021), and assembly (Berlin et al. 2015) of long-read sequencing data. With the widespread practical use of subsampling methods, several alternative subsampling techniques have been proposed to subsample *k*-mers with as low a density as possible (Marçais et al. 2018; Zheng et al. 2020), to subsample *k*-mers more evenly with weighted minimizers (Jain et al. 2020a, 2020b), and to subsample *k*-mers context independently with syncmers (Edgar 2021) or minimally overlapping words (Frith et al. 2020). In work by Shaw and Yu (2021), the investigators studied both theoretical and empirical performance of subsampling methods in detail.

Because of the error rates of long-read sequencing, the size of *k* chosen for subsampling methods is often much shorter (∼13–15 nt) than what is considered to produce mostly unique *k*-mers in, for example, the human genome (around *k* > 20 nt). With this length of minimizers, they produce many candidate sequence matches. Therefore, it would be useful to combine the robustness of minimizers to indels and mutation errors with larger *k*-mer sizes that would offer more unique matches. One approach is to use a small *k*-mer size and identify pairs (Altschul et al. 1997) or groups (Noé and Kucherov 2004) of them clustered tightly together, and it has been studied how to design the sampling distribution of seeds to optimize alignment sensitivity (Buhler et al. 2005; Sun and Buhler 2005). Multiseed methods are robust to any mutation type and have been shown to, for example, improve overlap detection between long reads (Du et al. 2019). However, these methods still match single *k*-mers individually and group them based on statistics after individual *k*-mer hits have been found. To remove the redundancy in matches, we suggest that it is beneficial to couple the *k*-mers before the matching step in order to perform a single constant-time lookup of coupled *k*-mers. Coupled *k*-mers have been explored by, for example, Chin and Khalak (2019) and Sahlin and Medvedev (2021), where *paired minimizers* are generated and stored as a single hash. A paired minimizer match signals that the region is similar between sequences. Because of the gap between the minimizers, such a structure is not as sensitive to indels or substitutions as *k*-mers. Paired minimizers were shown to be useful for both genome assembly (Chin and Khalak 2019) and error correction of long cDNA reads (Sahlin and Medvedev 2021) where the reads are similar only in some regions owing to alternative splicing. However, in work by both Chin and Khalak (2019) and Sahlin and Medvedev (2021), minimizers are produced independently and paired up after the minimizer generation. As mutations can alter the minimizers in a region, paired minimizers are sensitive to differences in the minimizer landscape. This holds true not just for pairing minimizers but for pairing *k*-mers from any subsampling method.

In this study, we propose a novel type of seed that we call a *strobemer*. Strobemers aims to address some of the limitations of previous seeding techniques. Unlike spaced *k*-mers, strobemers can match over indels, and unlike paired minimizers, they are not as sensitive to regions in which the minimizer landscape is different. Furthermore, compared with other seeding approaches that can match over indels (Giladi et al. 2010), only a single seed needs to be constructed per position in the sequence. Strobemers are, therefore, both fast and memory-efficient to construct.

## Results

### Strobemers overview

To construct a *k*-mer somewhere from a biological sequence *s*, one extracts *k* consecutive letters in *s*. Consequently, a *k*-mer only needs the length of the substring, *k*, as a parameter to be specified. A strobemer can be seen as a set of linked *k*-mers. Strobemers consist of *n* smaller ℓ-mers (*strobes*), where each ℓ-mer is extracted from a specified window in *s*. The first strobe is chosen at a fixed position in *s*, that is, the position we want to sample the strobemer from. For the consecutive strobes, lower (*w*_min_) and upper (*w*_max_) offsets to the previous strobe's window define the window in which the next strobe is extracted. Therefore, we parameterize a strobemer as (*n*, ℓ, *w*_min_, *w*_max_). The novelty compared with, for example, *k*-mers and spaced *k*-mers, is that strobemers allow flexibility in the strobes’ spacing and can produce matches between two sequences in a region with indels. There are different methods to produce the strobes, giving different results in performance for sequence matching and construction time. We explore three such methods here, called minstrobes, randstrobes, and hybridstrobes. The formal definition and construction of the strobemers are discussed in detail in the Methods section. The name *strobemers* is inspired by strobe sequencing technology (an early Pacific Biosciences sequencing protocol), which would produce multiple subreads from a single contiguous fragment of DNA in which the subreads were separated by “dark” nucleotides whose identity was unknown, illustrated by Ritz et al. (2010). Strobemers introduced here are, however, produced computationally.

### Strobemers in relation to other seeding techniques

We will compare strobemers to spaced *k*-mers and *k*-mers, which extract one subsequence per position from the sequence. Therefore, these methods (1) have a similar memory requirement to store reference seeds and (2) require only a single lookup per query seed position. Because of these requirements, we refer to spaced *k*-mers and strobemers as *k*-mer replacement methods (term used by Shaw and Yu 2021). Such methods differ from other seeding techniques, which require that multiple subsequences are stored and queried at each position (Burkhardt and Kärkkäinen 2002; Giladi et al. 2010; Lederman 2013). In addition, *k*-mer replacement methods are orthogonal to seeding techniques based on *k*-mer subsampling (e.g., minimizers, syncmers, and minimally overlapping words), which can be applied to subsample *k*-mers or *k*-mer replacement methods. Finally, the paired minimizer approach (Chin and Khalak 2019) relates closely to minstrobes with two strobes. The paired minimizer approach produces a subset of minstrobes, as minstrobes sample the first strobe at each position. This means that a minstrobes created with parameters (2, 15, *w*_min_, *w*_max_) provides an upper bound on the matching metrics obtained with paired minimizers sampled with a window size of (*w*_max_ − *w*_min_).

### Experimental overview

We will first investigate sequence matching performance of strobemers compared to *k*-mers and spaced *k*-mers using simulated data. We consider both how effective the different protocols are at finding matches under different error rates (related to sensitivity) and how unique the matches are that they produce (related to specificity).

We then implement a tool, StrobeMap, and use synthetic and biological data to show the utility of strobemers in various applications. We map Oxford Nanopore Technologies (ONT) cDNA reads with a 7% median error rate from Sahlin and Medvedev (2021) both to themselves and to reference sequences. We also map genomic ONT *Escherichia coli* reads with a 17% median error rate both to themselves and to an *E. coli* genome, as well as two *E. coli* genomes to themselves. In the experiments, we compare the contiguity and coverage of the matches produced by *k*-mers and strobemers.

### Experiment design

The size of the extracted subsequence length *k* of any protocol is central when comparing the efficacy of finding matches and their uniqueness. Therefore, we are interested in comparing sizes of subsequences that are similar between the protocols. Specifically, if the size of the *k*-mer is 30, we want to compare the *k*-mers to strobemers parameterized, for example, by (2, 15, ·, ·) and (3, 10, ·, ·), as all the extracted subsequences have a length of 30 on the strings. We similarly compare *k*-mers and strobemers to spaced *k*-mers where *k* positions are fixed but at different densities denoted sparse and dense (for details, see Methods).

### Evaluation metrics

If a *k*-mer or spaced *k*-mer extracted from position *i* in *s* and *i*′ in *t* produces the same hash value, we say that a *match* between two sequences, *s*_1_ and *s*_2_, occurs at positions *i* and *i*′ in the two strings, respectively. For a *k*-mer, we say that the match produces a *sequence coverage* over positions [*i*, *i* + *k*]. For a spaced *k*-mer, we say that the match produces a *sequence coverage* over the *k* fixed (sampled) positions. Furthermore, for a *k*-mer we say that the match has a *match coverage* of length *k* (i.e., positions [*i*, *i* + *k*]), and of length *L* in case of the spaced *k*-mer (i.e., the span of the fixed positions). If a strobemer extracted from position *i* in *s* and *i*′ in *t* produces the same hash value, we say that a match between two sequences, *s*_1_ and *s*_2_, occurs at position *i* and *i*′ as well as at the start positions of the additional strobes, *m*_2_, …, *m*_*n*_, in the two strings, respectively. We say that the match produces a *sequence coverage* over all the positions covered by the strobes in the match. Furthermore, we say that the match has a *match coverage* spanning the first nucleotide in the first strobemer to the last nucleotide in the last strobemer. The total sequence coverage and match coverage of a string, *s*, is calculated as the union of all positions covered under the definitions of sequence coverage and match coverage, respectively. We adopt similar terminology as in the work by Blanca et al. (2021) and denote a maximal interval of consecutive positions without matches as an *island*.

To evaluate the sequence matching ability, we compare under different error rates (1) the fraction of matches, (2) the sequence coverage, (3) the match coverage, and (4) the distribution of islands. We need to make two clarifications on these evaluation metrics. First, our experiments on simulated data are designed with parameters so that the event of observing a false match (e.g., repetitive *k*-mer) under any protocol has a negligible probability. This means that our simulated experiments only measure the raw ability to identify correct matches.

Second, as for the distribution of islands, we are interested in measuring the sizes of islands and their size distribution. We calculate the island *E-size* (Salzberg et al. 2012), a commonly used metric in genome assembly that we will adapt for our purposes. For a string, *s*, and a set of islands lengths, *X*, on *s* we calculate the island E-size, *E*, as follows:

$$E=\frac{1}{\left|s\right|}\underset{x\in X}{\sum }x^{2}.$$

*E* measures the *expected island size*, and intuitively, we can think of *E* as follows. We pick a position at random across *s* and observe the island size spanning that position. We may pick positions that are covered by matches (i.e., island size zero), but if we keep picking positions at random over *s* and store our observations on the island lengths, we will end up with *E* according to the *law of large numbers*. We will also show the entire island distribution.

### Strobemer versus *k*-mer matching

We compare how effective the different protocols are at producing matches for different error rates. We start with a controlled scenario, in which mutations are distributed with a fixed distance. In our second experiment, we use a random mutation distribution. We perform the fixed-distance mutation experiment to illustrate the advantage of strobemer protocols.

#### Controlled mutations

**Figure 1.**
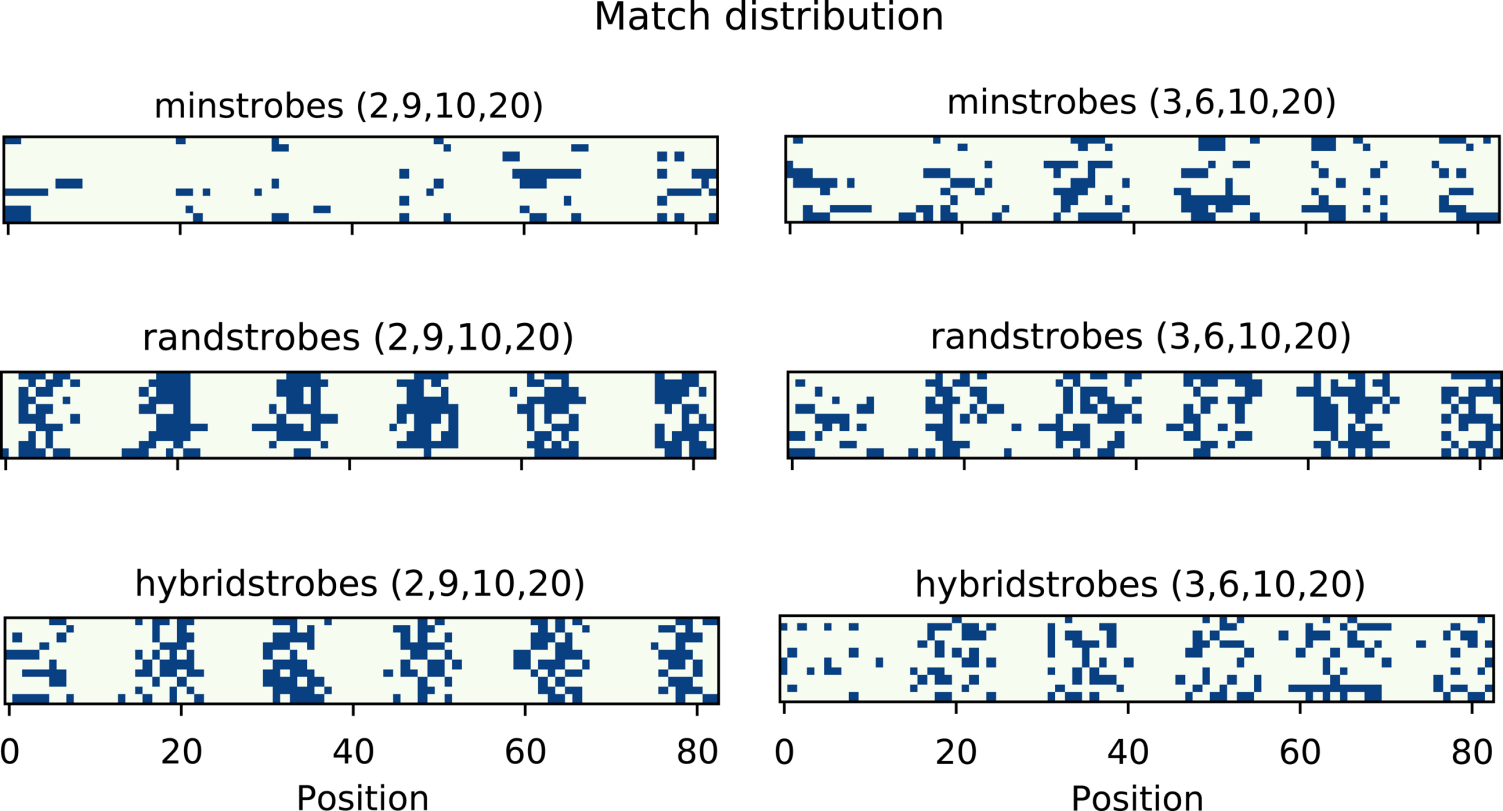
An example of strobemer matches for minstrobes, randstrobes, and hybridstrobes with two different parameterizations each (separate panels). Each panel shows matches between a string, *s*, of 100 nt and a string, *t*, derived from simulating mutations every 15th position in *s*. Indels and substitutions are chosen at random with equal probability. The matches are plotted with respect to the positions in *s* on the 83 possible matching positions (*x*-axis). Each row in a panel corresponds to a separate simulation.

We simulate a string, *s*, of 100 random nucleotides and a string, *t*, derived from simulating mutations every 15th position in *s*. The mutation is chosen as an inserted, deleted, or substituted nucleotide randomly with an equal probability of 1/3 each. We simulate *s* and *t* 10 times to illustrate the variability in matches for the strobemers between simulations. In this experiment, we chose the parameters of the strobemers so that their total length is 18, which is larger than 15, rendering *k*-mers and spaced *k*-mers of the same length infeasible, because there is a substitution or indel every 15 positions. The start positions of matching strobemers are shown in Figure 1 under two different parameterizations for minstrobes and randstrobes. Minstrobes, although more effective than *k*-mers in this scenario, fail to produce matches between many of the mutations for the (2, 9, 10, 20) parameterization and for some with the (3, 6, 10, 20) parameterization. We observe that randstrobes produce matches in all 10 experiments under both parameterizations and provide a more random match distribution across the string than minstrobes. Hybridstrobes have a match performance in between minstrobes and randstrobes.

#### Random mutations

In our second experiment, we simulate a string of length 10,000 nt and construct a second string by generating inserted, deleted, or substituted nucleotides with equal probability of 1/3 each across the string with the mutation rate *μ* ∈ 0.01, 0.05, 0.1. This means that the positions for the mutations are randomly distributed. Each such simulation is replicated 1000 times to alleviate sample variation. We refer to this as the SIM-R experiment (for simulation random). In this scenario, spaced *k*-mer protocols perform worse than *k*-mers, with fewer matches, lower match coverage, and larger expected island size (Table 1). We observe that *k*-mers have the highest fraction of matches in all experiments. This is because matches produced by *k*-mers cluster optimally tight (1-nt offset) between neighboring mutations at a distance larger than *k*. The minstrobe protocols under the two parameterizations have roughly the same performance as *k*-mers, with higher match coverage and smaller expected island size but a lower fraction of matches and sequence coverage. The randstrobe protocols are also in this scenario significantly better at distributing matches across the sequences compared to all the other protocols. The randstrobe protocols have a substantially higher sequence and match coverage, and smaller expected island size under both parameterizations, which are all important aspects of sequence matching. Hybridstrobes produce results that are relatively close to the performance of randstrobes across the four matching metrics.

**Table 1.**
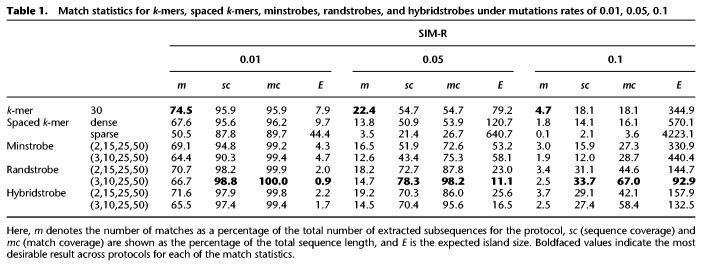
Match statistics for *k*-mers, spaced *k*-mers, minstrobes, randstrobes, and hybridstrobes under mutations rates of 0.01, 0.05, 0.1

We also show the full distribution of island sizes for the three different mutation rates for a subset of the protocols (Supplemental Fig. S1), which illustrates the general trend in island sizes. For example, for a mutation rate of 0.1, we observe that the randstrobe protocols have ∼1000 nt as the largest island size in our simulations, whereas *k*-mers have ∼2000 nt (Supplemental Fig. S1).

#### Subsampling

*k*-mers, spaced *k*-mers, and strobemers can all be subsampled using subsampling methods such as minimizers (Roberts et al. 2004), syncmers (Edgar 2021), or minimally overlapping words (Frith et al. 2020). We compared the protocols when applying a minimizer protocol with window sizes *w* = 10 and 20 to both sequences in the SIM-R experiments. For *k*-mers and spaced *k*-mers, the subsampling is performed by selecting the *k*-mer with the lowest hash value in a window of size *w*. For strobemers, the subsampling is performed by selecting the first strobe with the lowest hash value in a window of size *w*. This start strobe will be used to construct the full strobemer. In case of ties in hash values, the first *k*-mer (strobe) is selected.

In this scenario, the relative improvement of strobemers compared with *k*-mers decreases as *w* increases. For *w* = 10, randstrobes has a better sequence coverage, match coverage, and expected island size than all other protocols across mutation rates (Supplemental Table S1). With *w* = 20, *k*-mers produce the best sequence coverage across protocols, whereas randstrobes produce the best match coverage across protocols. Expected island size is better for randstrobes for mutation rates 0.01 and 0.05 but is worse for a mutation rate of 0.1. Hybridstrobes follow the performance of randstrobes closely in all experiments. Our experiments indicate that, under the subsampling method considered here, the relative increase in performance that strobemers have over *k*-mers decreases as sample size decreases.

### Strobemer versus *k*-mer uniqueness

Although preserving matches under mutations is an important feature for any seeding strategy, the other aspect is whether the constructed seeds are unique. Uniqueness is beneficial as any downstream algorithm will spend less time on evaluating spurious similarities caused by repetitive seeds. The importance of providing unique seeds for sequence comparison applications has been shown by significant work on algorithms and data structures to efficiently find unique sequences (Delcher et al. 1999; Haubold et al. 2005; Jain et al. 2020a).

**Figure 2.**
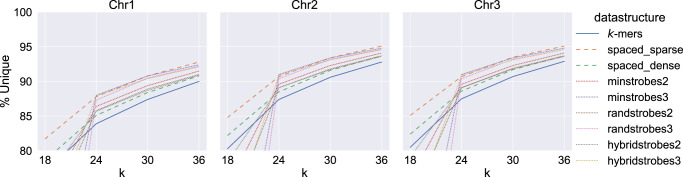
The percentage of unique *k*-mers, spaced *k*-mers, minstrobes, randstrobes, and hybridstrobes (*y*-axis) on the three largest chromosomes (Chr 1–Chr 3) of the human genome for various sequence lengths of *k* (*x*-axis). Each panel shows a separate chromosome. For a given *k* in the plot, strobemers with *n* = 2 are computed with parameters (2, *k*/2, *k*/2+1, 50) and strobemers with *n* = 3 are computed with parameters (3, *k*/3, *k*/3+1, 25) so that the numbers of extracted nucleotides between the five methods are the same. *Y*-axes have been cut at 80% for illustration. The values for minstrobes and randstrobes with parameters (3, 6, 7, 25) are below 50% on all the three chromosomes. The values for minstrobes and randstrobes with parameters (2, 9, 10, 50) are below 70% on all the three chromosomes.

Strobemers offer more match flexibility than *k*-mers and spaced *k*-mers, as they can preserve a match over indels in the sampled region. We refer to the ability for a protocol to match over indels as *flexible-position* protocols), contrary to *k*-mers and spaced *k*-mers (referred to as *fixed-position* protocols). It is reasonable to assume that for the same size *k* of extracted subsequence, the strobemer protocols will have lower uniqueness (precision) than *k*-mers and spaced *k*-mers owing to the flexible-position feature. We study the uniqueness in matches by computing the percentage of unique *k*-mers, spaced *k*-mers, and strobemers on the three largest human chromosomes (Fig. 2). For a *k*-mer size of *k*, we parameterize the strobemer protocols as (2, *k*/2, *k*/2+1, 50) and (3, *k*/3, *k*/3+1, 25) in order to have the same subsequence lengths as *k*-mers and spaced *k*-mers, and similar sampling span between the order 2 and 3 strobemers. Similarly, the spaced *k*-mers are parameterized by *L* = 1.5*k* and *L* = 3*k*, and the positions are simulated as in previous experiments (for details, see Methods subsection “Spaced *k*-mer sampling”).

We observe that for the three fixed-position protocols, a larger fixed-positions span helps subsequence uniqueness. The spaced-sparse has the highest uniqueness across all the three chromosomes, followed by spaced-dense and finally the *k*-mers.

Contrary to our intuition, strobemers offer a higher uniqueness than *k*-mers for *k* ≥ 24 (Fig. 2), which may be owing to the larger sampling window span, similarly to what we observed for the spaced *k*-mers. Out of the strobemer protocols evaluated here, strobemers of order 3 produce the highest percentage of unique matches for reasonably large subsequence lengths (*k* ≥ 24). There is no substantial difference between the strobemer protocols of the same order. However, for *k* = 18, the strobemer protocols will be parameterized by (2, 9, 10, 50) and (3, 6, 7, 25), which, with the flexible-position sampling, appear too small to guarantee reasonable uniqueness on the largest human chromosomes.

### Time and memory to construct strobemers

We compared construction time of strobemers to *k*-mers in both Python and C++ and observed very different results between strobemers in the two languages. We first compared the relative runtime of computing *k*-mers compared with strobemers in Python for different *k*-mer and window sizes (Supplemental Table S2). The *k*-mers are the fastest to compute. Randstrobes have the slowest relative runtime compared with *k*-mers, where the relative increase in computation time depends on the window size. Both minstrobes and hybridstrobes have comparable relative construction times to *k*-mers (Supplemental Table S2), making hybridstrobes, with their beneficial sequence match metrics, the most attractive protocol out of the strobemers. Therefore, in a scripting language, the construction time largely corresponds with our expectations that computing time is roughly proportional to window size.

In our C++ library, we implemented *k*-mers, minstrobes of order two, randstrobes of order two and three, and hybridstrobes of order two and three. We optimize the computation speed of *k*-mers and strobes by representing a nucleotide with two bits and using a sliding bit-packing window (64-bit integer) over the sequence. This technique avoids repetitive hashing of substrings to integers and is used in, for example, minimap2 (Li 2018). Because of the bit-packing, our implementation is limited to a maximum *k*-mer size of 32 and a maximum strobemer size of 32*n* for strobes of order *n*. We computed the wall-clock time to construct and push *k*-mers and strobemers to a vector in C++ (Supplemental Table S3). For the strobemers, we used window sizes of 20, 40, 60, 80, and 100. Overall, we observed that (1) computing and storing randstrobes are only about 1.5 − 2.5× slower than computing and storing *k*-mers, (2) computing randstrobes is more efficient than computing hybridstrobes for most window sizes in our implementation, and (3) the difference in computation time for randstrobes is negligible for window sizes of 20–100. From these observations, we hypothesize that the compiler is very efficient at computing randstrobes, because it does not require any overhead of a queue data structure (used for minstrobes and hybridstrobes) and because the window in which the minimum value is computed is a contiguous block in a vector that may, most of the time, be in the cache. It may therefore be advantageous to use randstrobes over hybridstrobes in a compiled language. Construction time of strobemer may also be further improved using, for example, single instruction multiple data (SIMD) implementations as is commonly used in bioinformatics (e.g., Daily 2016; Vaser et al. 2017).

The peak memory to compute and store *k*-mers and strobemers is similar if only the start position is stored. If all the positions of the strobemers are stored, the allocated memory will be higher. If we represent a *k*-mer with a 64-bit integer hash value, the reference id, and the position of the *k*-mer with 32 bit integers each, the memory occupied by the array storing *m k*-mers and their start position will be 128*m* bits (16*m* bytes). Strobemers have identical memory constraints, but if we decide to store the second and third position of the strobe, they will take an additional 4 bytes per strobemer unless stored as an offset to previous strobe.

### Proof-of-concept sequence mapper

As shown in our simulated experiments, spaced *k*-mers perform suboptimally to *k*-mers and strobemers when indels are present. Therefore, we further compared *k*-mers to strobemers using synthetic and biological data with indels. We implemented the proof-of-concept tool StrobeMap in both C++ and Python. The Python implementation of StrobeMap implements a sequence similarity search with *k*-mers and strobemers of order 2 and 3 without limitations to the *k*-mer and strobemer size, whereas the C++ implementation is limited to *k*-mers of size 32 or less and strobemers of size 32*n* or less, where *n* is the number of strobes. The output of StrobeMap is a tab-separated value (TSV) file with mapping information on the same format as MUMmer (Kurtz et al. 2004). However, instead of producing maximal exact matches (MEMs) or maximal unique matches (MUMs) between a query and a reference sequence, StrobeMap outputs what we refer to as nonoverlapping approximately matching (NAM) regions based on matches from the strobemer or *k*-mer protocol. The NAMs are produced by matches that overlap both on the query and reference; details on how NAMs are produced are found in Supplemental Note A.

As sequence mapping is often used as a preprocessing step to performing alignment or clustering, we use metrics valuable to candidate filtering to evaluate the methods. We measured the number of NAMs generated, the total match coverage produced by the NAMs, and the average normalized NAM length, which is the length of the NAM divided by either the length of the reference or the query depending on the mapping context. To achieve high accuracy and efficient sequence similarity searches, it is important that a mapping step produce few but long matches that cover a large portion of the query and/or the reference. Few matches will reduce time to postcluster matches and reduce disk space (if matches are stored), whereas long contiguous matches will improve the decision on whether a candidate matching region should be aligned or not. We mapped ONT cDNA and DNA reads with 7% and 17% median error rates both to reference sequences and to the reads themselves. We also studied whole-genome mapping of two *E. coli* genomes under some different settings. The details of the data and experiments are found in Supplemental Note A.

**Figure 3.**
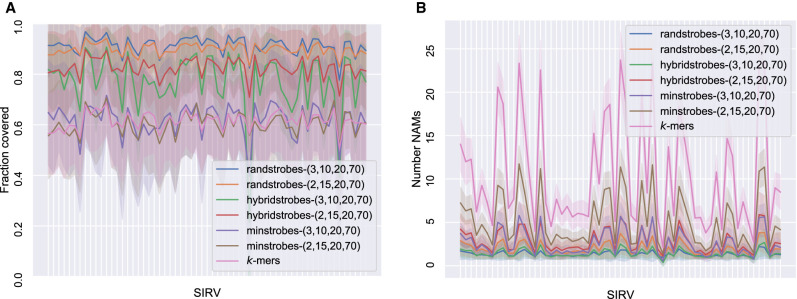
Comparison between strobemers and *k*-mers when matching ONT cDNA reads (7.0% median error rate) to 61 unique *spike-in RNA variant* (SIRV) reference sequences. Each SIRV corresponds to a tick on the *x*-axis. (*A*) Total fraction of the SIRV covered by NAMs from reads (*y*-axis). (*B*) The number of NAMs (*y*-axis) between a read and the SIRV. The line shows the mean, and the shaded area displays the standard deviation of the reads. A high NAM coverage and low number of NAMs means long contiguous matches and facilitates accurate and efficient sequence comparison.

We first mapped cDNA reads (queries) to SIRVs (references) using *k*-mers and strobemers with a subsequence size of 30, where strobemers were parameterized as (2, 15, 20, 70) and (3, 10, 20, 70). Randstrobes produce the highest match coverage to references (Fig. 3A), lowest number of matches (Fig. 3B), and highest normalized NAM lengths (Supplemental Fig. S2). On this data set, randstrobes are favorable to all other protocols when it comes to sequence matching, closely followed by hybridstrobes. Many of the NAMs that the randstrobes produce cover nearly the full SIRV reference (Supplemental Fig. S2). We observe the same trend when we compare the ability match reads from the same SIRV to each other (Supplemental Fig. S3). However, all the protocols produce a lower coverage and normalized match length owing to the lower sequence identity.

When mapping genomic ONT *E. coli* reads to an *E. coli* genome, we measure how many NAMs the protocols generate and the fraction of the read that is covered by NAM matches for the best mapping location. To get the best mapping location, we compute the longest collinear chain of NAMs to the genome. We count only the coverage of the longest collinear chain of NAMs to avoid overestimating coverage from additional matches owing to, for example, matches in repetitive regions. However, a collinear chain may still contain spurious matches. We, therefore, also measure the fraction of a NAM's genomic span that overlaps with the true genome mapping and refer to this overlap as correct. A NAM can have a correct overlap fraction between zero and one, where the fraction is zero if the match is fully outside the true mapping location and one if the match is fully within the true mapping location. We estimate the true genome mapping location from minimap2's primary alignments (for experiment details, see Supplemental Note A).

**Figure 4.**
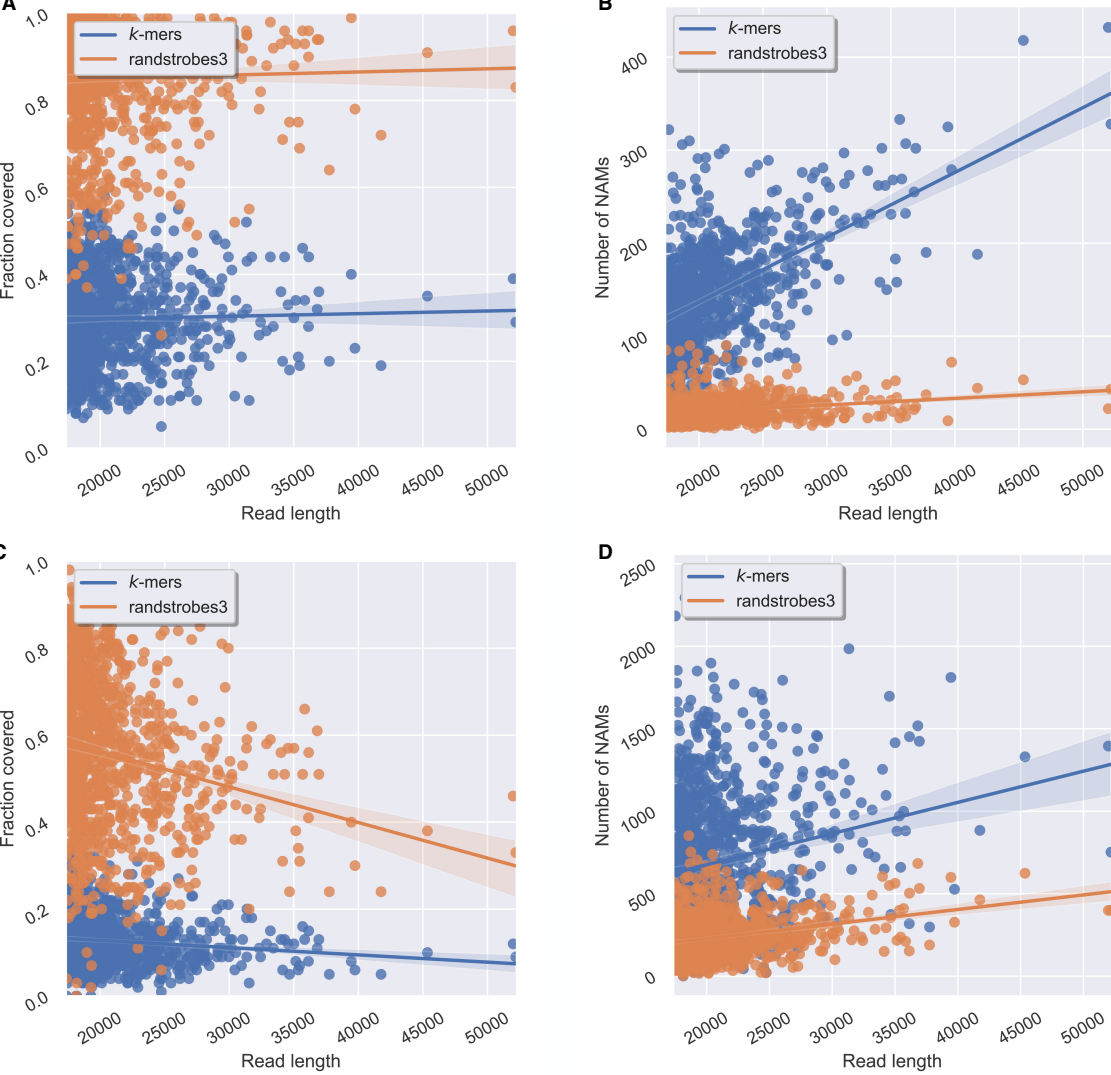
Comparison between randstrobes and *k*-mers when mapping genomic ONT reads for reads of different lengths (*x*-axis). Panels *A* and *B* show read mapping results when mapping reads to the genome, and *C* and *D* when mapping reads to themselves. (*A*) Total fraction of the read covered by NAMs in the optimal collinear chaining solution to the genome (*y*-axis). (*B*) The total number of NAMs (*y*-axis) between a read and the genome. (*C*) Total fraction of the read covered by NAMs to the longest overlapping read, inferred from the optimal solution of a collinear chaining (*y*-axis). (*D*) The total number of NAMs (*y*-axis) generated for the read. The line shows the mean, and the shaded area displays a 95% confidence interval of the mean estimate. High NAM coverage and low number of NAMs mean long contiguous matches and facilitate accurate and efficient sequence comparison.

We compared *k*-mers of length 30 to randstrobes with parameters (3,10,11,100). The NAMs produced by randstrobes cover more of the read (Fig. 4A) and are fewer (Fig. 4B). As for correctness, randstrobes and *k*-mers give 963 and 959 reads with a correctness of 1.0, respectively. The correctness fraction is shown for all reads in Supplemental Figure S4A. We also mapped the reads against themselves, and similarly to mapping to the genome, we computed the total number of NAMs as well as the coverage of the optimal collinear chain solution. Using only the coverage contributed from the matches in the collinear chain solution means that the coverage is only calculated for the largest overlap to another read. Similarly to when mapping reads to the genome, we measure correctness. We then measure the fraction of correctness of NAMs between reads from the true overlap between the reads based on minimap2's read-to-genome alignments (for details, see Supplemental Note A). For the read-to-read alignments, we observe that randstrobes produce higher NAM coverage (Fig. 4C) and fewer NAMs (Fig. 4D). We also observe that for randstrobes as seeds, 895 out of 1000 reads have a correctness of 1.0, whereas only 759 reads have a correctness of 1.0 using *k*-mers as seeds. The correctness fractions for the read-to-read mappings are shown in Supplemental Figure S4B.

StrobeMap and MUMmer are not competing tools as they produce different outputs. Nevertheless, at a high level, they are both mapping tools for finding local shared regions between sequences. Therefore, in addition to comparing sequence matching statistics between strobemers and *k*-mers, which is the primary intent with StrobeMap, we also included a comparison between StrobeMap and MUMmer (Table 2). We used comparable parameter settings between the tools and computed runtime, memory usage, and various match statistics when finding all shared regions between two *E. coli* genomes to each other and Chromosome 21 from hg38 to Chromosome 21 from CHM13 (Nurk et al. 2021) (for details, see Supplemental Note A). As can be inferred from the match *E*-size, (Table 2) StrobeMap produces longer contiguous NAMs with randstrobes compared with the NAMs produced with *k*-mers as well as the MEMs and MUMs produced by MUMmer. The increase in contiguity is not surprising as strobemers are designed to match over smaller indels and substitutions.

**Table 2.**
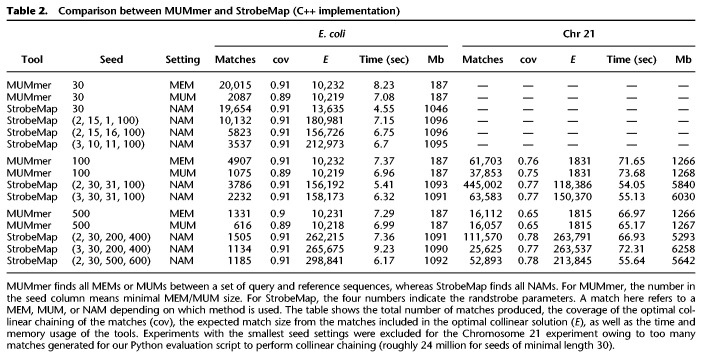
Comparison between MUMmer and StrobeMap (C++ implementation)

**Figure 5.**
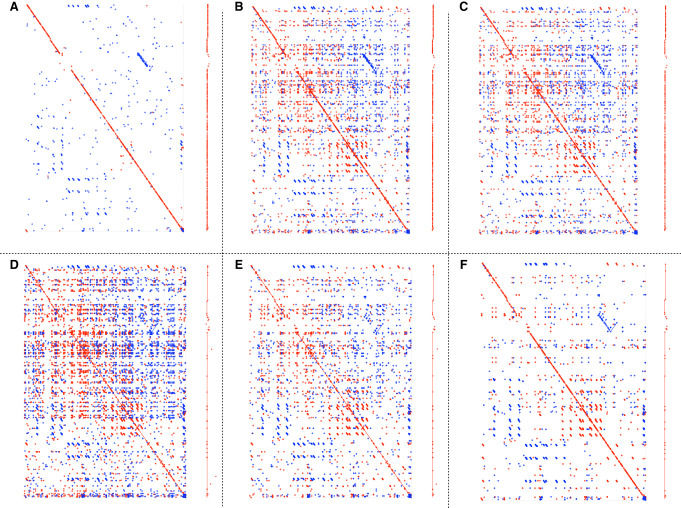
Dotplots and the optimal collinear chain solution produced by MEMs, MUMs, and NAMs from mapping two different *E. coli* genomes to each other. Each panel shows dotplots of all matches (*left*) and the optimal collinear chaining solution produced by the matches (*right*). The chaining solution has been put in vertical position for display. The panels show MUMmer with MUMs of a minimum size of 30 (*A*), MUMmer with MEMs of a minimum size of 30 (*B*), StrobeMap with *k*-mers of a size 30 (*C*), StrobeMap with (2,15,1,100) randstrobes (*D*), StrobeMap with (2,15,16,100) randstrobes (*E*), and StrobeMap with (3,10,11,100) randstrobes (*F*).

Figure 5 shows dotplots and optimal collinear chaining solutions for the matches produced for various settings for the *E. coli*–to–*E. coli* mapping experiment in Table 2. By design, MUMs (Fig. 5A) and MEMs (Fig. 5B) produce fragmented matches in the collinear chaining solution as they break at every mismatch. We also observe that the dotplot showing all MEMs of minimum size 30 (Fig. 5B) is nearly identical to the plot created with StrobeMap using *k*-mers of size 30 (Fig. 5C). Such a similar result is expected as NAMs produced from *k*-mers are nearly identical to MEMs, except that NAMs do not break over homopolymer and short tandem repeat differences. When using randstrobes, both small local NAMs and large contiguous NAMs can be preserved by specifying a small *w*_min_ values, as can be seen from comparing the dotplot to the contiguity of the collinear chaining solution (Fig. 5D). Many small NAMs can be removed by specifying a larger *w*_min_ (Fig. 5E,F).

### Time and memory usage of StrobeMap

The C++ implementation of StrobeMap represents the seed (i.e., *k*-mer or strobemer) as a 64-bit integer hash value, the reference accession as a 32-bit integer, and the position of the seed as a 32-bit integer as tuples in a vector sorted by seed hash value. If *k*-mers are used as seeds, only the start position is stored. If strobemers are used as seeds, the positions of the first and last strobe are stored. StrobeMap also uses a hash table with seed hash value as a key and the position and occurrence count of the seed in the sorted vector. The peak memory depends on the number of unique *k*-mers or strobemers and how many hits they generate in the mapping stage. In practice, we see a relatively small difference in peak memory usage for different seeds (Table 2). StrobeMap is as fast or faster than MUMmer for most settings. However, MUMmer has about a 6 × smaller peak memory usage compared with StrobeMap. StrobeMap is only a proof-of-concept tool to implement the experiments in this study. Reducing memory and runtime can be achieved by using, for example, subsampling methods such as minimizers.

## Discussion

We have studied strobemers, an alternative sampling protocol to *k*-mers and spaced *k*-mers for sequence comparison. We have experimentally shown that strobemers, particularly randstrobes and hybridstrobes, efficiently produce higher sequence coverage, match coverage, and lower gap size between matches under different mutation rates (Table 1). Strobemers also produce a higher number of unique matches (specificity) compared with *k*-mers for several commonly used sizes of *k* (Fig. 2).

As *k*-mer matches cluster optimally tight between mutations at distance larger than *k*, they produce the highest number of matches in the SIM-R experiments. However, the number of matches is not always helpful as matches may cluster owing to local repeats. Randstrobes and hybridstrobes can offer more evenly distributed matches, higher match coverage, and higher uniqueness. These are features that are useful for several algorithms that require chains of matches between two sequences to be considered candidates for alignment, clustering, or read overlap detection (e.g., Li 2016, 2018; Chin and Khalak 2019; Sahlin and Medvedev 2020).

As for time to generate strobemers, we observe that randstrobes are only about 1.5 − 2.5× slower to generate and store than *k*-mers in our compiled implementation (Supplemental Table S3), whereas they are significantly slower to generate in script languages such as Python (Supplemental Table S2). As randstrobes has the best performance in terms of sequence comparison according to our analyses, we recommend them over minstrobes and hybridstrobes in most sequence comparison applications implemented in compiled languages. In scripting languages, hybridstrobes have the best trade-off in performance and sequence comparison accuracy (Supplemental Table S2).

To further show the utility of strobemers, we implemented a mapping tool, StrobeMap, for finding local matches between sequences using both *k*-mers and strobemers. We showed in several different scenarios, such as mapping ONT cDNA and genomic reads to themselves or to reference transcripts or genomes, that strobemers produce favorable sequence comparison metrics compared with *k*-mers (Figs. 3, 4). Although StrobeMap primarily was implemented as a proof-of-concept tool for our experiments, it is as fast or faster than MUMmer in the majority of our genome-to-genome mapping experiments, albeit with a substantially higher peak memory usage (Table 2). Particularly, hybridstrobes offer a beneficial trade-off between construction time and the ability to produce long contiguous matches under various sequence matching contexts. Overall, we believe that strobemers offer a valuable contribution to methods that rely on sequence similarity search.

### Future study of strobemers

#### Parameterization

Although our study provides an experimental evaluation of strobemers under some commonly used values of *k* and mutation rates, the statistics of strobemers remain to be explored. In work by Blanca et al. (2021), the investigators derived the mean and variance of islands for *k*-mers and the number of mutated *k*-mers under given mutation rate. If we can derive analytic expressions for strobemers, it may suggest to us how to optimize parameters of the strobemer protocols under various mutation rates, which will be useful for similarity comparison algorithms. Even without analytic expressions, we can evaluate the sizes on strobes and windows suitable for various mutation rates. Also, we could relax the constraint of equal-size strobes and window sizes. As a start in this direction, we may derive more efficient parameter selection on window sizes by modeling the number of mutations after a certain number of nucleotides as a Poisson process. Under such a model, the investigator hypothesizes that choosing larger window sizes downstream could be beneficial. This remains to be explored.

#### Structural variation detection

Strobemers are currently designed to match only over substitutions and small indels. It remains as future work to investigate if larger windows or alternative designs can span over larger variants. For example, one could design strobemers with different window ranges between, for example, two chromosomes, to detect larger indels. Another example would be to detect inversion breakpoints by constructing strobes with different directions, for example, one forward and one reverse complement.

#### Construction, storing, and queries

There are several aspects of construction, indexing, and storage of strobemers that could be explored. One such direction is to store and query the positions of the other strobes efficiently, as they give extra information about the coverage and span of matches across sequences for sequence similarity applications. Another application is to efficiently index the data sets for abundance and presence of strobemers (Marchet et al. 2020). For such applications, minstrobes may be advantageous owing to the more frequently shared minimizers between the strobes. Finally, the possibility of further decreasing practical runtime for constructing strobemers remains to be explored.

#### Span-coverage for matching

Because strobemers are gapped sequences, it also motivates the study of match coverage and distribution of matches across regions (or positions), similarly to what has been performed for gapped experimental protocols such as mate-pair or paired-end reads (Sahlin et al. 2017). For example, one could compute the span-coverage of matches at positions or over regions to estimate the sequence similarity in matching regions or the confidence for further downstream processing.

#### Subsampling

We observed that, under our subsampling approach, the more sparsely the strobemers are subsampled, the less advantage they have over *k*-mers (Supplemental Table S1). An interesting future research direction would be to study whether specific subsampling schemes are better suited for strobemers: specifically, whether they can preserve the relative performance increase that are observed without thinning. By studying the mathematical properties of hashes and minimizers (Orenstein et al. 2017; Marçais et al. 2018), we may find an effective subsampling method for strobemers.

#### Generalization of strobemers

We can view the process of extracting a *k*-mer or a spaced *k*-mer at position *i* in a string, *s*, as applying a function *f*(*i*, *k*, *s*) on *s*. Similarly, the process of extracting a strobemer from *s* can be viewed as applying the higher-order function *f*′(*i*, *k*, *s*, *h*) on *s*, where *h* is either some hash function or hash strategy (e.g., iterative and conditionally dependent as in randstrobes). We showed that applying *f*′ on *s* is equally or more efficient than *f* for sequence matching for three different functions *h* (minstrobes, randstrobes, and hybridstrobes), which poses the following question. Can we further improve sampling protocols for sequence matching by designing *h* differently?

## Methods

### Definitions

We refer to a *subsequence* of a string as a set of ordered letters that can be derived from a string by deleting some or no letters without changing the order of the remaining letters. A substring is a subsequence in which all the letters are consecutive. We use *i* to index the position in a string *s* and let *s*[*i* : *i* + *k*) denote a *k-*mer substring at position *i* in *s* covering the *k* positions *i*, …, *i* + *k* − 1 in *s*. We will consider one-indexed strings. If *s*[*i*: *i* + *k*) is identical to a *k*-mer *t*[*i*′ : *i*′ + *k*) in string *t*, we say that the *k*-mers *match* and that the match occurs at position *i* in *s* (and *i*′ in *t*). Similarly, let *f*(*i*, *k*, *s*) be any function to extract a subsequence of length *k* with first letter at position *i* from *s*. If *f*(*i*, *k*, *s*) is identical to *f*(*i*′, *k*, *t*), we say that the subsequences match and that the match occurs at position *i* in *s* (and *i*′ in *t*). For example, for *k*-mers we have *f*(*i*, *k*, *s*) = *s*[*i* : *i* + *k*). We let |·| denote the length of strings.

We use *h* to denote a hash function $h:\sum^{*}\to Z$, mapping strings to numbers. Given the two integers *w* > 0 and *k* > 0, the minimizer at position *i* in *s* is the substring of *s* of length *k* starting in the interval of [*i*, *i* + *w*) that minimizes *h*.

### Aim

We will introduce strobemers by describing the problem they aim to solve. Consider two strings, *s* and *t*, that are identical up to *m* mutations. We desire a function, *f*, to produce a set of subsequences from *s* and *t* that have two characteristics: (1) There should be as few as possible placements of the *m* mutations that result in no matches between *s* and *t*, and (2) the subsequences of length *k* should be as unique as *k*-mers on *s* and *t*. Characteristics 1 and 2 relate to match sensitivity and precision, and we will discuss this in an example below. For practical purposes, we also require that at most one subsequence is produced per position to mimic how *k*-mers are derived in a string (and limit the amount of data we store for each string). Certainly, we could produce all possible subsequences at each position to minimize criteria 1, but this is not feasible. A similar objective to characteristic 1 was studied for multiseed design (Sun and Buhler 2005), in which the investigators wanted to find a set of seeds so that at least one seed matched a gapless alignment between two sequences.

### A motivational example

Consider two strings of 100 nt with *m* = 3 mutations between them. This could occur, for example, in splice alignment to an exon or in sequence clustering. If we use a *k*-mer of size 30 to find matches and if the two strings differ at positions 25, 50, and 75, there will be no matching *k*-mers. Similarly, this holds for mutations at positions 20, 48, and 73 and several other combinations. As described, we want as few possible placements of errors leading to the region being unmatched.

Using spaced *k*-mers (Ma et al. 2002) or permutations of the string (Lederman 2013) would help if the mutations were substitutions. We could consider lowering *k*, but this would generate more matches to other strings as well. To achieve the same uniqueness as longer *k*, we could consider coupled *k*-mers (Altschul et al. 1997) of say 15 nt per pair, with some gap in between them. Note that the *k*-mers would need to be coupled before searching for matches to avoid many matches to other sequences. Furthermore, if the coupled *k*-mers have a fixed distance from each other, we have just created a specific type of spaced *k*-mers, which are only robust to substitutions. We, therefore, could consider coupled minimizers (Chin and Khalak 2019; Sahlin and Medvedev 2021) to select a random gap size for us, but in a deterministic manner.

This brings us to the strobemers. In the scenario above, we could pick a *k*-mer of size 15 at a position we want to sample and couple it with a minimizer of length 15 derived from a window downstream from the *k*-mer. Together, they have sequence length 30 and are therefore robust to false matches. They can also match across the mutations, where the mutations could be both substitutions and indels. If we increase the mutation density on our string, eventually, our two *k*-mers of length 15 nt will also fail to produce any matches. Therefore, we could consider triplets of a *k*-mer and two minimizers of length, for example, 10 nt. Finally, we can further reduce the sampled minimizers’ dependency, and therefore the positional correlation of the matches, using other hashing protocols (as we will investigate here).

### Strobemers

Consider a string, *s*. A strobemer of order *n* in *s* is a subsequence of *s* composed of a set of ordered substrings *m*_1_, …*m*_*n*_ on *s* of equal length ℓ, that we call *strobes*. If the first strobe, *m*_1_, starts at position *i*, the second strobe, *m*_2_, will be selected from a downstream window [*i* + *w*_min_ : *i* + *w*_max_] with *w*_min_ < *w*_max_. Similarly, *m*_3_ will be selected downstream from *m*_2_ in the window [*i* + *w*_min_ + *w*_max_ : *i* + 2*w*_max_] on *s*. To generalize, strobe *m*_*n*_ (*n* > 1) is selected from the region [*i* + *w*_min_ + (*n* − 2)*w*_max_ : *i* + (*n* − 1)*w*_max_] in *s*. We will from now on parameterize a strobemer as (*n*, ℓ, *w*_min_, *w*_max_), denoting the order, the length of the strobe, and the minimum and maximum window offset to the previous window, respectively.

**Figure 6.**
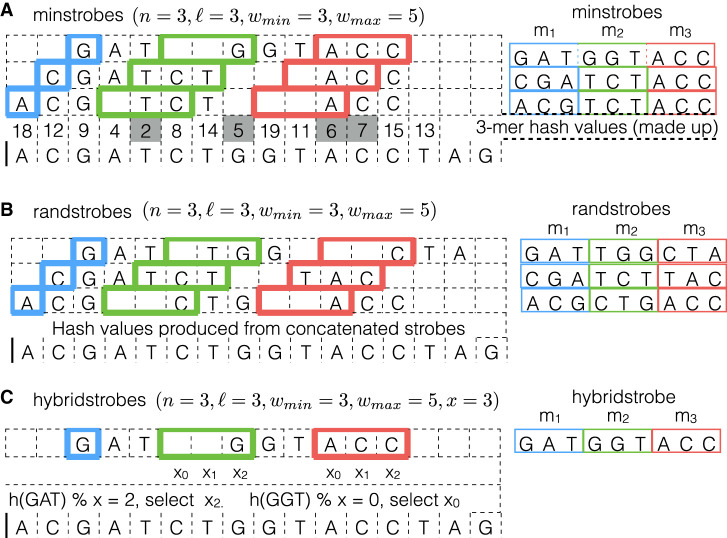
An illustration of three minstrobes (*A*) and three randstrobes (*B*) with (*n* = 3, ℓ = 3, *w*_min_ = 3, *w*_max_ = 5), and one hybridstrobe (*C*) with (*n* = 3, ℓ = 3, *w*_min_ = 3, *w*_max_ = 5, *x* = 3) generated from a DNA string of 16 letters. With parameters *n* = 3 and ℓ = 3, the strobemers will consist of three strobes (substrings) each of length 3. The position of the first strobe, *m*_1_, in each of the strobemers is highlighted in blue. The rest of the strobemers are chosen from a window of *w*_max_ − *w*_min_ + 1 = 3 positions based on the minimizer method of minstrobes (*A*), randstrobes (*B*), or hybridstrobes (*C*). The possible start positions of strobes *m*_2_ and *m*_3_ are highlighted in green and red, respectively. For the minstrobe method *A*, the 3-mer minimizer hash values (under a made up hash function in the figure) are shown *above* the DNA string and come from computing *h*(*m*) for each 3-mer strobe *m*. The position of the hash value corresponds to the first position of the 3-mer strobe. The minimizer values in all relevant strobe windows of length 3 in the figure are indicated by gray squares. For the minstrobe method, strobes *m*_2_ and *m*_3_ are selected independently based on the minimizer value in each strobemer window. This gives a high similarity between nearby strobemers (sharing minimizers). The three minstrobes produced are shown to the *right* in *A*. For the randstrobe method *B*, strobes *m*_2_ and *m*_3_ are selected depending on the previous strobes, namely, *h*(*m*|*m*_1_, …, *m*_*i*−1_). The function producing the conditional dependence is irrelevant for the purpose of illustration. Here we use string concatenation of previous strobes to produce the dependence, but any other function producing conditional dependence will suffice. Because of the conditional dependence in the hash function, randstrobes are more randomly (but deterministically) distributed across the sequence. For the hybridstrobe method *C*, strobes *m*_2_ and *m*_3_ are selected from one of the *x* subwindows depending on the remainder of the previous strobe. Each subwindow has individually computed minimizers similar to the minstrobes. However, allowing the sampling of a strobe from one of the *x* windows to depend on the remainder of the previous strobe creates more sampling variability than minstrobes.

We will describe three different methods for computing the strobemers, which produce different results in terms of sequence match metrics and computing time. First, we denote as *minstrobe*, a strobemer in which strobes *m*_2_, …, *m*_*n*_ are minimizers in their respective windows under any hash function, *h* (Fig. 6A). That is, a minstrobe consists of a starting strobe concatenated with *n* − 1 consecutively concatenated minimizers.

Second, we denote as *randstrobe*, a strobemer in which strobe *m*_*j*_ is selected dependent on the previous *m*_1_, …*m*_*j*−1_ strobes (Fig. 6B). Consider the function $h(m^{\prime }|m_{1},\ldots ,m_{\;j-1})=h(m_{1}⊕\ldots \;⊕m_{\;j-1}⊕m^{\prime })$, where ⊕ denotes string concatenation, and let *m*_*j*_ be the *m*′ ∈ [*i* + *w*_min_ + (*j* − 2)*w*_max_ : *i* + (*j* − 1)*w*_max_] that minimizes *h*. That is, previously selected strobes influence the decision of *m*′ as opposed to the minstrobes method. We have here abstracted over *h* as any hash function taking a string as input and producing an integer. There are several ways to implement the conditional dependence *h*(*m*′|*m*_1_, …, *m*_*j*−1_) more efficiently. An example for compiled languages is to represent the strobes as binary numbers and to perform bit operations to produce the strobes. Specifically, let *t*_*j*_ denote the binary number representation of strobe *m*_*j*_ obtained by converting the letters A, C, G, and T to the two-bit representations 00, 01, 10, and 11, and let & denote the bitwise AND operator. Then we can compute $h(t^{\prime }|t_{1},\ldots ,t_{\;j-1})=((t_{1}+\ldots +t_{\;j-1}+t^{\prime })\&q)$, where *q* is a bitmask consisting of ones at the 16 lowest-order bits and remaining zeroes, and select the *t*′ = *t*_*j*_ that minimizes *h*. Such an operation is efficient in compiled languages. Finding the most efficient method to compute randstrobes is subject for future work.

Third, we will consider a hybrid between minstrobes and randstrobes that uses both independent minimizers and a conditional dependence between strobes that we call *hybridstrobes* (Fig. 6C). Consider partitioning the sampling window for each strobe into *x* disjoint segments of length $w_{x}=\lfloor (w_{\max}-w_{\min})/x\rfloor$. That is, the sampling window for *m*_2_ is partitioned into *x* disjoint subwindows [*i* + *w*_min_ : *i* + *w*_min_ + *w*_*x*_), [*i* + *w*_min_ + *w*_*x*_ : *i* + *w*_min_ + 2*w*_*x*_), …[*i* + *w*_min_ + (*x* − 1)*w*_*x*_ : *i* + *w*_max_), and similarly for the sampling windows of the other strobes. We select a strobe, *m*_*j*_, as the minimizer in the *r*th window segment of length *w*_*x*_ dependent on the remainder, *r*, of the previous strobe modulo, *x*, namely, $r=h(m_{\;j-i})\;\mathrm{mod}\;x$. In this study, we use *x* = 3.

An important aspect of the three protocols is the randomness in sampling of the strobes. For two strobes with nearby starting positions, strobes *m*_2_,.., *m*_*n*_ will most frequently be the same under the minstrobe generation owing to independent minimizers (see Fig. 6) and will most frequently differ in a randstrobe. This means that under the same parameters in the protocols, the randstrobes will (in all likelihood) contain more uniquely sampled positions and, hence, more unique randstrobes, whereas minstrobes more frequently share minimizers. Hybridstrobes place somewhere in between minstrobes and randstrobes depending on the size of *x*. Although the strobe selection is not as random as randstrobes, having *x* possible candidates for each strobe produces more randomly distributed matches than minstrobes.

We have described how to produce the individual strobes for the three protocols, but we did not mention yet how to represent the actual strobemers. Similarly to *k*-mers, the representation of strobemers in memory is flexible and depends on the application. For example, the concatenated strobes could be stored as a string representing the actual sequence of a strobemer. Alternatively, we could store a hash value representing the strobemer. Similar to *k*-mers, if a hash value is stored representing the strobemer, the specific hash function dictates the rate of hash collisions leading to false-positive matches. We discuss the details of how we represent a strobemer in the Implementation section.

We also note that minstrobes of order 2 are similar to but formally different from paired minimizers (Chin and Khalak 2019; Sahlin and Medvedev 2021). Both minstrobes of order 2 and paired minimizers consist of two substrings. However, paired minimizers are two minimizers that are coupled together under some distance constraint on a sequence, whereas for minstrobes, the first strobe is not necessarily a minimizer. Therefore, paired minimizers of length ℓ with distance constraint *w*_max_ are a subset of minstrobes produced with parameters (2, ℓ, 1, *w*_max_).

### Construction of strobemers

We aim to produce strobemers of a string, *s*, in a similar manner to how *k*-mers are produced, namely, one strobemer per position, *i* ∈ [1, |*s*| − *k* + 1]. This would mean that we extract the same amount of *k*-mers and strobemers from a string, *s*, and, consequently, for equal length *k*, the same amount of raw data. Note, however, that the number of unique *k*-mers and strobemers may differ. We construct strobemers as follows. The total possible span of a strobemer of order *n* is *W* = (*n* − 1)*w*_max_ + ℓ, and the total subsequence length is $k=\sum_{\;j=1}^{n}m_{j}$ with *w*_min_ ≥ ℓ (no strobe is overlapping). Let us consider extraction of a strobemer at position *i* in *s*. If *W* ≤ |*s*| − *i*, we use the predefined windows [*w*_min_, *w*_max_) and compute the strobemers under the respective strobemer protocols as described above. If *W* > |*s*| − *i*, we narrow the window sizes until *m*_1_ to *m*_*n*_ are all adjacent to each other, producing a substring (*k*-mer) of length *k*. Under this construction, the same amount of *k*-mers and strobemers will be extracted from a string. Here, we choose to shorten each window [*w*_min_, *w*_max_] to ⌊|s|−i⌋/n.

For applications such as read mapping, narrowing the window at the end of the read may result in different strobemers being extracted from the end of the read and the reference. Therefore, the narrowing of windows at the end of a sequence can be omitted from such applications. Furthermore, the protocol to extract strobemers allows overlapping strobes with *w*_min_ < ℓ. For example, a strobemer with parameterization (2, 15, 1,70) may produce strobemers that span between 16 and 85 nt on the sequence, counting the span as the leftmost to the rightmost nucleotide in the strobemer. We will here consider *w*_min_ ≥ ℓ giving nonoverlapping strobes, assuring that $\sum_{\;j=1}^{n}m_{j}=k$. The pseudocode to construct strobemers is given in Supplemental Note B.

### Time complexity

If we ignore the time complexity of the hash function, the time complexity of generating minimizers is *O*(|*s*|*w*) for window size *w*. However, as previously noted (Li 2016), computing minimizers is in practice close to *O*(|*s*|) if we use a queue to cache previous minimizer values in the window. The expensive step is when a previous minimizer is discarded from the queue and a new minimizer needs to be computed for the window.

Similar to computing minimizers, strobemers have a worst-case time complexity of *O*(|*s*|*n*(*w*_max_ − *w*_min_)). However, the independence of hash values in the minstrobe and hybridstrobe protocols makes them close to *O*(|*s*|) in practice by using separate queues for each strobe sampling window in the same manner as computing minimizers independently. The randstrobe protocol does not have this independence under the hashing scheme we consider in this study, which means that all hash values have to be recomputed at each position. This means its practical time complexity is therefore *O*(|*s*|*n*(*w*_max_ − *w*_min_)).

### Implementation

The pseudocode to construct strobemers (Supplemental Note B) is provided for the simplicity in expression, they are not efficient implementations. We want to avoid string concatenation. We also want to avoid repeated computation of minimizers for minstrobes and hybridstrobes where minimizer values are computed independently.

We first precompute all the hash values in a string to work with addition of hash values instead of string concatenations. For minstrobes and hybridstrobes, we keep a queue data structure with the hash values in the current sampling window for each strobe *m*_*j*_, *j* ≥ 2 and the current minimum hash value in each window. This allows us to only recompute the minimum hash values in the window whenever we discard the current minimum in the queue, as described previously (Li 2016). For randstrobes, we cannot use queues. We instead select the strobe *m*′ in a window that minimizes the function $(t+t^{\prime })\&\;q$, where *t* and *t*′ are bit representations of the previous strobe *m* and the candidate strobe *m*′, respectively, and *q* is a bit-mask of 16 ones, and “&” is the bitwise AND operator.

For strobemers of order 2 consisting of strobes *m*_1_ and *m*_2_, the final strobemer hash value that is stored will be *h*(*m*_1_)/2 + *h*(*m*_2_)/3. We store the hash value to represent the strobemer and not the sequence of the strobemer explicitly. Similarly, for strobemers of order 3 consisting of strobes *m*_1_, *m*_2_, and *m*_3_, we store *h*(*m*_1_)/3 + *h*(*m*_2_)/4 + *h*(*m*_3_)/5. We divide each hash value for two reasons. First, it makes the function asymmetric. An asymmetric function should be used so that a permutation of the strobes within a strobemer do not produce the same strobemer hash value. Second, the division of each hash value ensures that there is no overflow when representing the hash value in a 64-bit integer.

### Spaced *k*-mer sampling

The spaced *k*-mers consist of a window of size *L* with *k fixed* positions and a set of *L* − *k* wildcard (or “don't care”) positions. This is commonly represented as a binary string in which ones are sampled and zeros are wildcard positions. For example, in the string *AGGTCA* with *L* = 6, the spaced *k*-mer 101011 is *AGCA*. In our evaluations, we choose two densities of fixed positions for the spaced *k*-mers. First, we denote as *spaced-dense* a strategy in which 2/3 of the positions are fixed and denote as *spaced-sparse* a strategy in which 1/3 of the positions are fixed. The spaced-dense and the spaced-sparse frequency of fixed positions roughly correspond to the densities used by Břinda et al. (2015) and Leinonen and Salmela (2021), respectively. To keep *k* fixed, we use *L* = 1.5*k* in the spaced-dense protocol and *L* = 3*k* in the spaced-sparse protocol. The windows’ first and last positions are always fixed (see Břinda et al. 2015; Leinonen and Salmela 2021) to assure the length of the spaced *k*-mer. The remaining fixed positions are randomly chosen. In, for example, the work by Leinonen and Salmela (2021), the sampled positions are handpicked. Although handpicking positions may be more suitable for optimizing lower correlation between matches, this study focuses on designing a protocol robust to indels. We will observe that spaced *k*-mers do not work well for mutations other than substitutions.

### Software availability

All the scripts used for the analysis and evaluation, as well as the Python and C++ reference implementations of our tool StrobeMap, together with installation and usage instructions, are available at GitHub (https://github.com/ksahlin/strobemers) and as Supplemental Code. The C++ version of StrobeMap used in this study is available at GitHub (https://github.com/ksahlin/strobemers/releases/tag/0.0.1). All scripts used for the analysis and evaluation are available at GitHub (https://github.com/ksahlin/strobemers/tree/main/evaluation).

## Supplementary Material

Supplemental Material
