# Supplementary material for: Effective sequence similarity detection with strobemers
Source: Genome Res. 2021 Nov;31(11):2080–94. doi: 10.1101/gr.275648.121 (PMC8559714; doi:10.1101/gr.275648.121)
Supplement: Supplemental Material [file supp_31_11_2080__DC1.html]

Effective sequence similarity detection with strobemers — Supplemental Material 

# Effective sequence similarity detection with strobemers

## Supplemental Material

- Supplemental\_Methods.pdf
- Supplemental\_code.zip
