# Supplementary material for: Effective sequence similarity detection with strobemers
Source: Genome Res. 2021 Nov;31(11):2080–94. doi: 10.1101/gr.275648.121 (PMC8559714; doi:10.1101/gr.275648.121)
Supplement: Supplemental Material [file supp_gr.275648.121_Supplemental_Methods.pdf]

Supplementary Material to
Effective sequence similarity detection with strobemers

Kristoffer Sahlin[1]

[1]Department of Mathematics, Science for Life Laboratory, Stockholm University, 106 91, Stockholm, Sweden.

Contents

A: Mapping analysis **1**

B: Strobemers construction **7**

E: Figures **10**

F: Tables **14**

A: Mapping analysis

Constructing Non-overlapping Approximate Matches (NAMs)

The NAMs are produced as follows. Assume a query sequence q and a reference sequence r, and two strobemers a and b where the match of a spans positions $[q_i, q_j]$ and $[r_i, r_j]$ and the match of b spans $[q_{i'}, q_{j'}]$ and $[r_{i'}, r_{j'}]$ on q and r, respectively. If it holds that $q_i \leq q_{i'} \leq q_j$ and $r_i \leq r_{i'} \leq r_j$ we say that they *overlap* where a precedes b and b supersedes a. A NAM spanning $[q_1, q_2]$ on the query sequence and $[r_1, r_2]$ on the reference is an ordered set of overlapping strobemers such that the NAM does not have a preceding or superseding overlap

with any other strobemer on both q and r. Note that there may however still be strobemers that overlaps with a NAM on either reference or the query, or even produce a preceding overlap on the query and a superseding overlap on the reference (or vice versa) due to repeat structure.

The definition of NAMs for k-mers is identical where a k-mer match, as opposed to a strobemer match, spans k consecutive positions. Note that NAMs produced by k-mers is not the same as a Maximal Exact Match (MEM) because homopolymer or satellite repeat differences that are longer than the k-mer will break a MEM but will not break a NAM.

StrobeMap

StrobMap is a proof-of-concept implementation for finding all NAMs between a set of query and reference sequences using either k-mers of strobemers. It is implemented for the experiments performed in his study. The input of StrobMap is two fasta files with reference and query sequences, and the output is a tab separated values file (TSV) file with mapping information on the same format as MUMmer [1], but containing all NAMs instead of MEMs or MUMs.

StrobeMap is implemented in both Python and C++. The C++ implementation uses bitpacking of nucleotides into two bits for more efficient computation and is limited to a strobe size ≤ 32. This means that maximum strobemer size is $32n$ for strobemers of order n. In the C++ implementation, k-mers, minstrobes of order 2, randstrobes of order 2 and 3, and hybridstrobes of order 2 and 3 are implemented. This implementation can be used as follows for producing, *e.g.*, randstrobes with $(3, 20, 21, 100)$

```
StrobeMap -n 3 -k 20 -v 21 -w 100 -c randstrobes
          -o matches.tsv
          references.fa queries.fa
```

The python implementation of StrobeMap implements sequence similarity search with k-mers and all strobemers of order 2 and 3 without limitations to the k-mer and strobemer size. The Pyhton implementation is slower and use more memory than the C++ implemen-

tation. This implementation can be used as follows for producing, *e.g.*, randstrobes with $(3, 20, 21, 100)$

```
StrobeMap --n 3 --k 20 --strobe_w_min_offset 21 -strobe_w_max_offset 100
          --rev_comp --randstrobe_index
          --queries  queries.fa
          --references references.fa
          --outfolder out/ --prefix matches
```

SIRV reads to SIRV references

We downloaded SIRV ONT cDNA reads from ENA with accession number PRJEB34849, and SIRV references from `https://www.lexogen.com/wp-content/uploads/2018/08/SIRV_Set1_Lot00141_Sequences_170612a-ZIP.zip`. We preprocessed the cDNA reads using pychopper (`https://github.com/nanoporetech/pychopper`) to produce full-length cDNA sequences. We then aligned the full-length reads using minimap2 [2] to the references and subsampled 100 reads from each reference. For each SIRV we subsampled from the pool of reads that had a primary alignment to the SIRV that started and ended not more than ten nucleotides from the start and end of the SIRV, respectively. This was done to assure that in an ideal matching scenario, a NAM from a read to a SIRV could cover the entire SIRV.

SIRV reads to each other

In this experiment, we took the 100 reads subsampled as described in the previous section, and mapped each of the 100 reads to the other 99 reads within the pool for each SIRV.

E. coli genomes to each other

We mapped the *E. coli* genome GCA_003018135.1 ASM301813v1 to the *E. coli* genome GCA_003018575.1 ASM301857v1 available at `https://www.ncbi.nlm.nih.gov/genome/167?genome_assembly_id=368391`.

We ran the C++ implementation of StrobeMap as follows

```
StrobeMap  -o outfolder \
          GCF_003018135.1_ASM301813v1_genomic.fna \
          GCF_003018575.1_ASM301857v1_genomic.fna
```

with the specific parameters

```
-k 30 -kmers
```

to produce k-mer NAMs and, for example,

```
-k 15 -v 16 -w 100 --n 2
```

to produce hybridstrobes with parameters (2,15,16,100).

Human chromosome 21 to each other

We mapped human chromosome 21 from hg38 to CHM13 [3] with StrobeMap as follows

```
/usr/bin/time -l StrobeMap -o tmp.tsv \
          chr21_hg38.fa \
          chr21_chm13.fa \
          2> runtime.txt
```

Where parameters n, k, v, and w was set according to the experiment parameter settings.

We ran MUMmer to find MEMs as follows

```
/usr/bin/time -l mummer -F -maxmatch -l 30 -b \
        chr21_hg38.fa \
        chr21_chm13.fa \
        > tmp.tsv 2> runtime.txt
```

and with MUMmer to find MUMs as follows

```
/usr/bin/time -l mummer -F -mum -l 30 -b \
        chr21_hg38.fa \
        chr21_chm13.fa \
        > tmp.tsv 2> runtime.txt
```

We computed the matching metrics, collinear solution, runtime, and memory using a script available in the strobemers repository on github. We ran the script as follows

```
python genome_mapping_metrics.py  tmp.tsv runtime.txt \
     --refs $genome2 --collinear_matches_out coll_sol.tsv
```

E. coli reads to E.coli genome

We downloaded *E. coli* reads from Sequence Read Archive with Run ID SRR13893500. As the sample contains a fraction of reads from other bacteria, we selected the 1000 longest reads from the sample that aligned to the *E. coli* genome with more than 95% of the total read length. As aligned portion we computed the span between the first and last base that was aligned to the genome divided by the read length. This calculation excludes hard and softclipped ends but does consider eventual poorly aligned internal regions of the read. This produced 1,000 reads with a median length of 19,601nt where the longest read was 52,197nt and the shortest read was 17,360nt. The total length of the reads was 21,020,364 giving a coverage of 6.65x. To obtain the subsampled reads, we ran minimap2 as follows and a custom script available in the strobemer repository to select the reads from the SAM file.

```
minimap2 -ax map-ont --eqx GCF_003018135.1_ASM301813v1_genomic.fna \
                            SRR13893500.fastq > SRR13893500.sam
python select_longest_reads.py SRR13893500.fastq SRR13893500.sam \
                                  1000 SRR13893500_1000_longest.fastq
```

We further estimated the read error rate from these reads by dividing the sum of substitutions and indels with the length of the aligned region to get a median error rate of 17.0%. We mapped the 1,000 reads using StrobMatch to the *E. coli* genome GCA_003018135.1 ASM301813v1 available at `https://www.ncbi.nlm.nih.gov/genome/167?genome_assembly_id=368373` and measured the number of NAMs and the match coverage produced by the colinear chain of matches that covers the largest fraction of the reads. We count the coverage only for the colinear chains as smaller matches to other region of the genome may inflate the coverage of the read to the actual best aligned region. However, all matches to the genome

contribute to the total number of matches, because this is an important efficiency metric to select candidate alignments.

E. coli reads to each other

We ran StrobeMap as follows

```
StrobeMap  -o outfolder \
           SRR13893500.fastq \
           SRR13893500.fastq
```

with the specific parameters

```
--k 30 --kmer_index
```

to produce k-mer NAMs and

```
--k 10 --strobe_w_min_offset 10 --strobe_w_max_offset 100 --n 3
```

to produce hybridstrobes with parameters (3,10,10,100).

Computing correctness of NAMs

We compute the fraction of a NAM's genomic span that overlaps with the true genome mapping location. A NAM match can have a fraction of correctness between 0 and 1. The correctness is 1.0 if the NAM is fully within the true genome mapping location and 0.0 if the NAM is fully outside the true mapping location. We estimate the true genome mapping location from minimap2's primary alignment. We align *E. coli* reads to an *E. coli* genome with minimap2 as follows

```
minimap2 -ax map-ont GCF_003018135.1_ASM301813v1_genomic.fna
        SRR13893500_1000_longest_reads.fastq >
        SRR13893500_1000_longest_reads_minimap2.sam
```

For each NAM in the longest collinear solution between a read and the genome, we then compute the fraction of the region spanned by the NAM on the reference that overlaps with the true mapping location obtained from minimap2. A NAM can have a fraction of correctness between 0 and 1. The total fraction of correctness of a read is obtained by summing over the NAM matches the total length of the correct regions divided by the total length of all the correct regions. For read to read overlaps, the fraction of correctness is computed in a similar manner. However, the true overlaps are here obtained from the true overlap between reads based on the genomic coordinates of reads to the genome from minimap2's alignments.

B: Strobemers construction

Algorithm 1: Minstrobes construction

Input: s, n, k, w_{min}, w_{max}

Output: Minstrobes of order n and their positions from s

S =[] # Initialize array of strobemers and their positions

$\ell = \lfloor k/n \rfloor$ # Strobe lengths

for $i \in [1, |s| - k + 1)$ **do**

  $w_u = min(w_{max}, (|s| - i)/(n-1))$ *# Second argument only active at end of s*

  $w_l = max(w_{min} - (w_{max} - w_u), \ell)$

  $m_1 = s[i : i + \ell)$

  $m = m_1$

  for $j \in [2, n]$ **do**

    $w' = [i + w_l + (j-2)w_u, i + (j-1)w_u]$ *# Window to look for current strobe*

    $p = \arg\min_p \{p : h(s[p : p + \ell)) \leq h(s[i' : i' + \ell)), \forall i' \in w'\}$

    m += *s[p:p+ℓ)* *# String concatenation*

  end

  S +=*(i,m)*

end

Algorithm 2: Randstrobes construction

Input: s, n, k, w_{min}, w_{max}

Output: Randstrobes of order n and their positions from s

$S = []$ # Initialize array of strobemers and their positions

$\ell = \lfloor k/n \rfloor$ # Strobe lengths

for $i \in [1, |s| - k + 1)$ **do**

  $w_u = min(w_{max}, (|s| - i)/(n-1))$ # *Second argument only active at end of s*

  $w_l = max(w_{min} - (w_{max} - w_u), \ell)$

  $m_1 = s[i : i + \ell)$

  $m = m_1$

  for $j \in [2, n]$ **do**

    $w' = [i + w_l + (j-2)w_u, i + (j-1)w_u]$ # *Window to look for current strobe*

    $p = \arg\min_p \{p : h(m \oplus s[p : p + \ell)) \leq h(m \oplus s[i' : i' + \ell)), \forall i' \in w'\}$

    m += $s[p{:}p{+}\ell)$ # *String concatenation*

  end

  S +=(i,m)

end

Algorithm 3: Hybridstrobes construction

Input: s, n, k, w_{min}, w_{max}

Output: Hybridstrobes of order n and their positions from s

$S = []$ # Initialize array of strobemers and their positions

$\ell = \lfloor k/n \rfloor$ # Strobe lengths

for $i \in [1, |s| - k + 1)$ **do**

  $w_u = min(w_{max}, (|s| - i)/(n-1))$ *# Second argument only active at end of s*

  $w_l = max(w_{min} - (w_{max} - w_u), \ell)$

  $w_x = (w_u - w_l + 1)//x$ *# Partitioned window lengths*

  $m_1 = s[i : i + \ell)$

  $m = m_1$

  for $j \in [2, n]$ **do**

    r = h(m) % x *# Compute residual*

    $w' = [i + w_l + (j-2)w_u + rw_x, i + (j-1)w_u + (r+1)w_x]$

      # Window to look for current strobe

    $p = \arg\min_p \{p : h(s[p : p + \ell)) \leq h(s[i' : i' + \ell)), \forall i' \in w'\}$

    m += s[p:p+ℓ) *# String concatenation*

  end

  S +=(i,m)

end

E: Figures

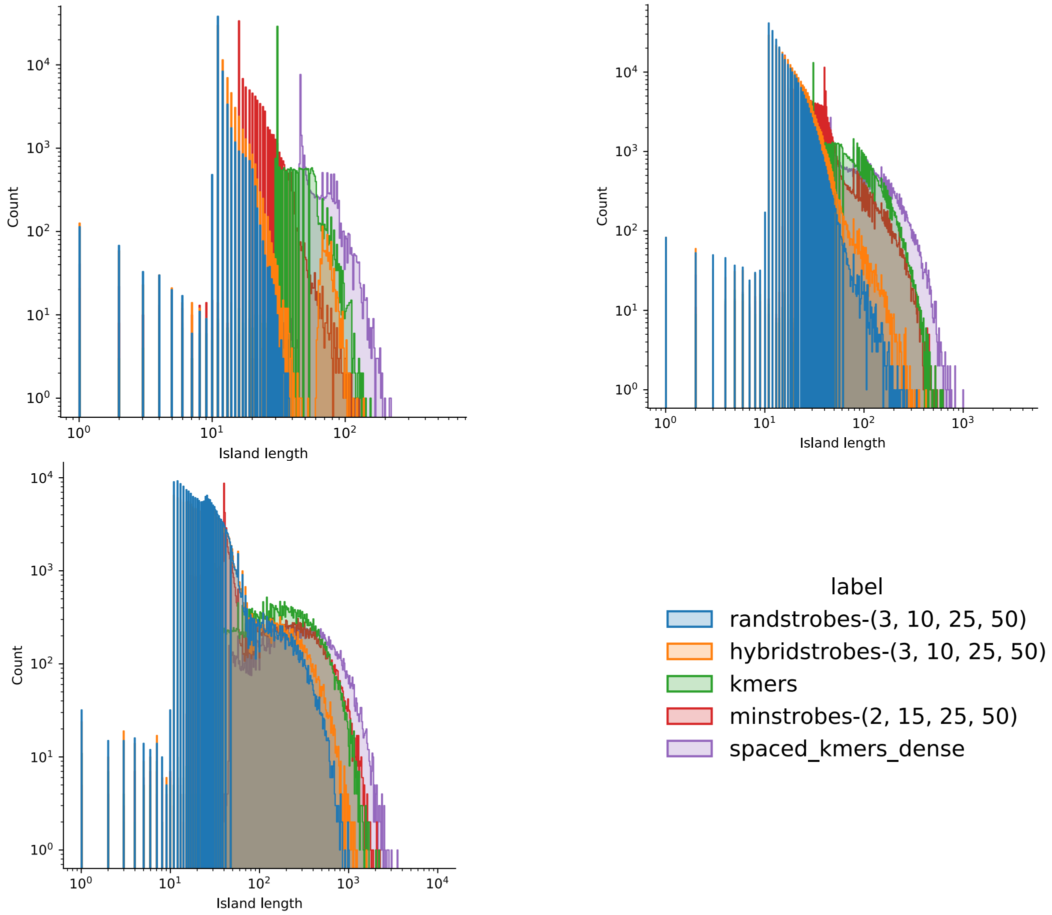

Figure S1: Histograms of island lengths for the SIM-R experiments for mutation rate 0.01 (A), 0.05 (B), and 0.1 (C).

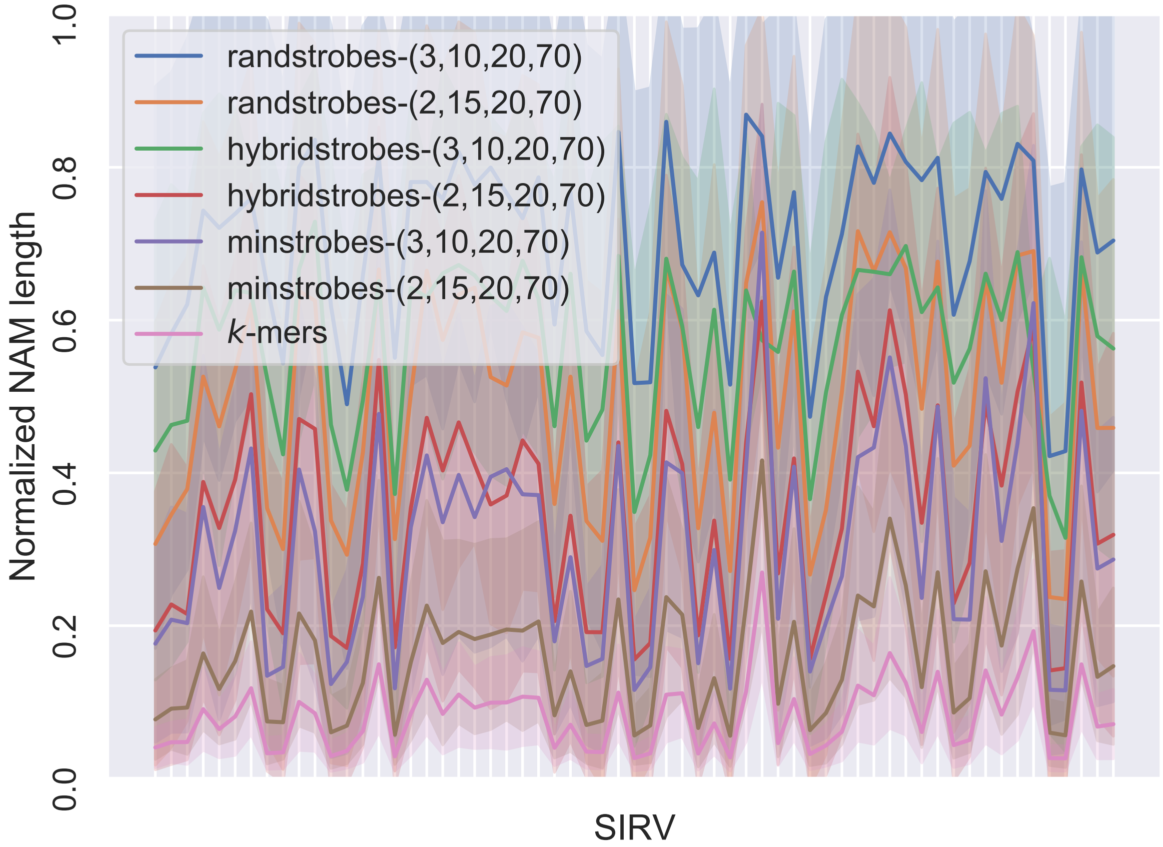

Figure S2: The plot shows the average normalized NAM length from all the NAM matches when matching ONT cDNA reads to 61 unique SIRV reference sequences. The normalized NAM length is the length of the NAM divided by the SIRV reference. Each tick on the x-axis corresponds to a SIRV. The line shows the mean and the shaded area displays the standard deviation of the reads. A high NAM coverage and low number of NAMs means long contiguous matches and facilitates accurate and efficient sequence comparison.

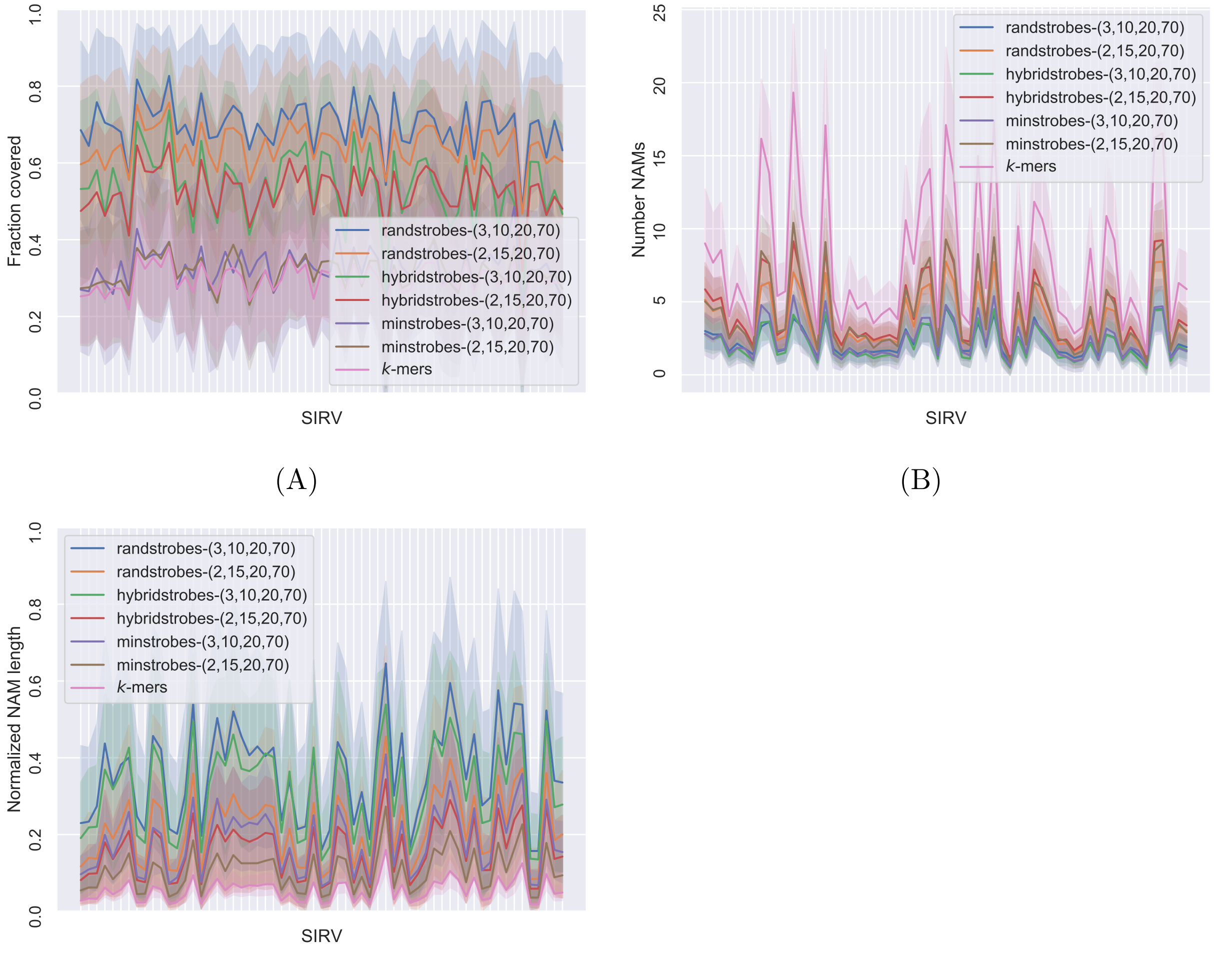

(A) (B)

(C)

Figure S3: Comparison between strobemers and k-mers when matching 100 ONT cDNA reads to themselves from each of the 61 SIRVs. For each reference SIRV, there are 9,900 sequence mappings as read self-mapping results, which produce perfect matches are excluded. Each tick on the x-axis corresponds to a SIRV. Panel **A** shows total fraction of reference reads covered by NAMs from query reads (y-axis). Panel **B** shows the number of NAMs (y-axis) between the query and reference reads reads. Panel **C** shows the average normalized NAM length from all the NAM matches. The normalized NAM length is the length of the NAM divided by the length of the read acting as the reference in the given match. The line shows the mean and the shaded area displays the standard deviation of the reads. A high NAM coverage and low number of NAMs means long contiguous matches and facilitates accurate and efficient sequence comparison.

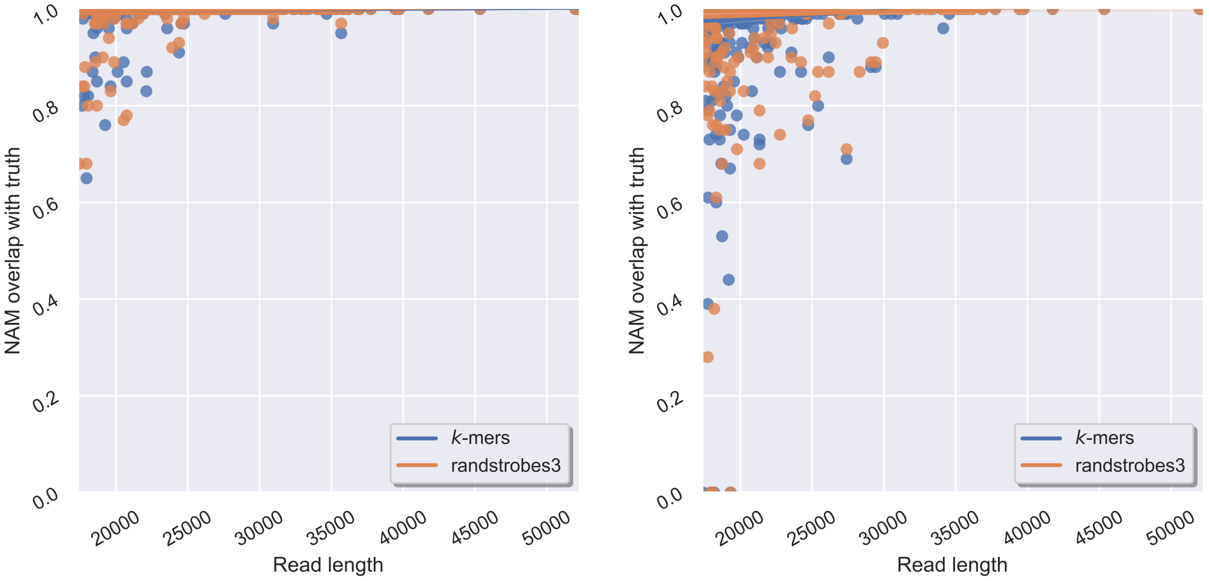

(A) (B)

Figure S4: Total fraction of the match coverage that overlap with true overlaps where ground truth is estimated from minimap2's alignments. Match coverage is produced from the longest collinear chain of NAM matches. Panel A) Mapping reads to E coli genome. Panel B) Read to read overlap detection.

F: Tables

			SIM-R											
			0.01				0.05				0.1			
			m	sc	mc	E	m	sc	mc	E	m	sc	mc	E
$w=10$	k-mer	30	**73.2**	90.2	90.2	14.9	**20.6**	42.6	42.6	115.2	**4.0**	11.6	11.6	524.2
	spaced k-mer	dense	65.5	87.1	90.7	22.2	12.1	30.7	36.3	215.3	1.4	5.4	7.0	1318.4
		sparse	47.9	74.4	84.7	72.3	2.8	9.6	16.6	1067.0	0.1	0.5	1.2	7197.4
	minstrobe	(2,15,25,50)	67.8	87.7	98.1	8.7	15.3	37.2	59.0	94.9	2.6	8.8	16.7	618.6
		(3,10,25,50)	63.3	81.2	98.4	8.9	11.6	28.7	60.1	116.2	1.7	5.9	16.2	926.0
	randstrobe	(2,15,25,50)	69.4	92.3	98.4	5.6	16.7	**45.6**	62.3	71.9	2.9	**11.7**	18.6	**475.4**
		(3,10,25,50)	65.6	**93.1**	**99.8**	**3.3**	13.6	43.8	**79.4**	**51.9**	2.1	9.7	**26.4**	507.6
	hybridstrobe	(2,15,25,50)	68.4	90.5	97.7	6.7	15.8	42.5	59.0	81.9	2.6	10.5	16.9	538.2
		(3,10,25,50)	62.0	88.2	99.0	6.0	11.8	37.0	72.9	71.2	1.8	8.0	22.5	628.3
$w=20$	k-mer	30	**71.9**	**84.2**	84.3	21.4	**19.3**	**33.1**	33.3	157.6	**3.7**	**7.7**	8.0	**769.4**
	spaced k-mer	dense	64.5	78.1	85.2	31.8	11.4	21.7	27.7	298.1	1.3	3.2	4.5	1984.8
		sparse	47.3	62.4	80.4	87.4	2.7	5.9	12.4	1430.7	0.1	0.3	0.7	8109.5
	minstrobe	(2,15,25,50)	66.7	75.8	95.2	18.8	14.3	25.9	46.0	156.7	2.4	5.3	10.8	1026.6
		(3,10,25,50)	62.2	59.6	95.9	20.3	10.8	18.5	46.1	209.0	1.6	3.4	10.1	1543.0
	randstrobe	(2,15,25,50)	68.3	83.9	94.8	12.1	15.7	31.3	46.1	130.2	2.6	6.5	10.9	847.5
		(3,10,25,50)	64.6	81.9	**99.1**	**8.8**	12.7	27.6	**61.1**	**115.9**	2.0	5.1	**15.1**	1002.0
	hybridstrobe	(2,15,25,50)	66.1	82.2	93.0	13.6	13.8	28.2	41.6	154.5	2.2	5.4	9.2	1003.6
		(3,10,25,50)	60.2	75.6	97.7	12.7	10.8	23.6	55.5	144.7	1.6	4.3	13.1	1180.6

Table S1: Match statistics under different sampling protocols under mutations rates of 0.01, 0.05, 0.1 using minimizer thinning with $w = 10$, and $w = 20$. Here, m denotes the number of matches as a percentage of the total number of extracted subsequences for the protocol, sc (sequence coverage) and mc (match coverage) is shown as the percentage of the total sequence length, and E is the expected island size.

		k-mers	minstrobes		randstrobes		hybridstrobes	
k	w		2	3	2	3	2	3
18	1	1.0	4.6	5.9	3.4	4.3	NA	NA
18	10	1.0	2.7	4.3	7.1	13.5	3.9	7.2
18	20	1.0	2.4	3.4	8.8	16.9	3.0	5.3
18	30	1.0	2.5	3.7	13.5	26.3	3.3	5.8
18	40	1.0	2.6	3.7	17.6	34.1	3.2	5.7
18	50	1.0	2.6	3.8	21.2	41.1	3.5	5.9
18	100	1.0	2.5	3.5	39.1	78.3	3.2	5.6
36	1	1.0	3.8	8.3	3.6	5.8	NA	NA
36	10	1.0	2.7	4.4	7.8	13.6	3.9	6.7
36	20	1.0	2.6	4.4	10.6	20.6	3.3	5.9
36	30	1.0	2.5	3.7	13.5	25.8	3.3	5.7
36	40	1.0	2.4	3.5	16.1	30.6	3.0	5.2
36	50	1.0	2.6	3.6	20.3	39.9	3.4	5.7
36	100	1.0	2.5	3.4	39.4	73.0	3.1	5.5
54	1	1.0	3.7	8.1	3.6	5.9	NA	NA
54	10	1.0	2.7	4.3	6.8	12.3	3.5	6.3
54	20	1.0	2.6	3.9	10.3	19.2	3.3	5.8
54	30	1.0	2.6	3.7	13.7	26.0	3.2	5.9
54	40	1.0	2.5	3.5	17.0	32.5	3.2	5.7
54	50	1.0	2.5	3.6	20.0	40.8	3.3	5.8
54	100	1.0	2.5	3.6	37.7	74.8	3.4	5.8
60	1	1.0	3.7	8.1	3.6	5.8	NA	NA
60	10	1.0	2.6	4.0	6.5	12.1	3.5	6.1
60	20	1.0	2.6	3.8	10.2	21.1	4.0	6.5
60	30	1.0	2.5	4.0	14.0	25.1	3.2	5.5
60	40	1.0	2.6	3.7	17.0	32.4	3.1	5.6
60	50	1.0	2.5	3.6	20.7	40.1	3.3	5.7
60	100	1.0	2.5	3.6	39.3	76.7	3.2	5.5
72	1	1.0	3.6	8.1	3.5	5.8	NA	NA
72	10	1.0	2.7	4.1	6.7	13.2	3.9	6.4
72	20	1.0	2.5	3.9	11.0	21.7	3.6	6.1
72	30	1.0	2.6	4.0	14.1	27.8	3.3	5.8
72	40	1.0	2.4	3.5	17.4	32.9	3.3	5.6
72	50	1.0	2.5	3.4	19.9	39.9	3.1	5.7
72	100	1.0	2.4	3.5	38.1	77.7	3.8	6.4

Table S2: Relative time to compute k-mers compared to strobemers of order 2 and 3 using python v3.8 for various subsequence sizes (k) and window sizes ($w = w_{max} - w_{min} + 1$). The computation time is normalized with the time to compute k-mers. Hybrid strobes are not defined for window sizes smaller than x (the number of partitions of each window), which we here have set to 3.

		E. coli	chr21 (hg38)	chr1 (hg38)
kmers	30	0.2s	1.7s	12.4s
minstrobes	(2, 15, 16, 36)	0.3s	2.3s	18.3s
minstrobes	(2, 15, 16, 56)	0.3s	2.5s	19.4s
minstrobes	(2, 15, 16, 76)	0.3s	2.3s	21.8s
minstrobes	(2, 15, 16, 96)	0.3s	2.2s	18s
minstrobes	(2, 15, 16, 116)	0.3s	2.3s	16.7s
hybridstrobes	(2, 15, 16, 36)	0.4s	2.8s	18s
hybridstrobes	(2, 15, 16, 56)	0.5s	3.6s	24s
hybridstrobes	(2, 15, 16, 76)	0.5s	3.9s	25.5s
hybridstrobes	(2, 15, 16, 96)	0.6s	4.4s	28.9s
hybridstrobes	(2, 15, 16, 116)	0.7s	4.9s	32.6s
randstrobes	(2, 15, 16, 36)	0.4s	2.9s	19s
randstrobes	(2, 15, 16, 56)	0.4s	3s	19.1s
randstrobes	(2, 15, 16, 76)	0.4s	3s	19.1s
randstrobes	(2, 15, 16, 96)	0.4s	3s	19.4s
randstrobes	(2, 15, 16, 116)	0.4s	3.1s	20.3s
hybridstrobes	(3, 10, 11, 31)	0.5s	3.8s	24.7s
hybridstrobes	(3, 10, 11, 51)	0.7s	4.8s	31.7s
hybridstrobes	(3, 10, 11, 71)	0.9s	6.1s	45.3s
hybridstrobes	(3, 10, 11, 91)	1.1s	7.4s	51.8s
hybridstrobes	(3, 10, 11, 111)	1.4s	9.3s	59.1s
randstrobes	(3, 10, 11, 31)	0.5s	3.4s	24s
randstrobes	(3, 10, 11, 51)	0.5s	3.6s	25.1s
randstrobes	(3, 10, 11, 71)	0.5s	3.6s	25.3s
randstrobes	(3, 10, 11, 91)	0.5s	3.5s	26.7s
randstrobes	(3, 10, 11, 111)	0.5s	3.8s	27.7s

Table S3: Time to compute k-mers compared to strobemers of order 2 and 3 for window sizes of size 20, 40, 60, 80 and 100 using a C++ implementation. The implementation uses bit packing of k-mers/strobes into 64 bit integers which significantly improves construction speed, but limits the implementation to k-mers of size 32 or less and strobemers of size $32n$ where n is the number of strobes. The timing includes computing the hash value representation of the k-mer (or strobemer) and pushing the hash value together with the position of the k-mer (positions of strobes) to a vector from the standard library using push_back method. The runtime is computed on *E. coli* (5.7Mb), human chromosome 21 (48Mb) and human chromosome 1 (248Mb).

Bibliography

References

[1] Kurtz, S., Phillippy, A., Delcher, A. L., Smoot, M., Shumway, M., An-

tonescu, C., and Salzberg, S. L. Versatile and open software for comparing large genomes. *Genome Biology 5*, 2 (2004), R12.

[2] Li, H. Minimap2: pairwise alignment for nucleotide sequences. *Bioinformatics 34*, 18 (05 2018), 3094–3100.

[3] Nurk, S., Koren, S., Rhie, A., Rautiainen, M., Bzikadze, A. V., Mikheenko, A., Vollger, M. R., Altemose, N., Uralsky, L., Gershman, A., Aganezov, S., Hoyt, S. J., Diekhans, M., Logsdon, G. A., Alonge, M., Antonarakis, S. E., Borchers, M., Bouffard, G. G., Brooks, S. Y., Caldas, G. V., Cheng, H., Chin, C.-S., Chow, W., de Lima, L. G., Dishuck, P. C., Durbin, R., Dvorkina, T., Fiddes, I. T., Formenti, G., Fulton, R. S., Fungtammasan, A., Garrison, E., Grady, P. G., Graves-Lindsay, T. A., Hall, I. M., Hansen, N. F., Hartley, G. A., Haukness, M., Howe, K., Hunkapiller, M. W., Jain, C., Jain, M., Jarvis, E. D., Kerpedjiev, P., Kirsche, M., Kolmogorov, M., Korlach, J., Kremitzki, M., Li, H., Maduro, V. V., Marschall, T., McCartney, A. M., McDaniel, J., Miller, D. E., Mullikin, J. C., Myers, E. W., Olson, N. D., Paten, B., Peluso, P., Pevzner, P. A., Porubsky, D., Potapova, T., Rogaev, E. I., Rosenfeld, J. A., Salzberg, S. L., Schneider, V. A., Sedlazeck, F. J., Shafin, K., Shew, C. J., Shumate, A., Sims, Y., Smit, A. F. A., Soto, D. C., Sović, I., Storer, J. M., Streets, A., Sullivan, B. A., Thibaud-Nissen, F., Torrance, J., Wagner, J., Walenz, B. P., Wenger, A., Wood, J. M. D., Xiao, C., Yan, S. M., Young, A. C., Zarate, S., Surti, U., McCoy, R. C., Dennis, M. Y., Alexandrov, I. A., Gerton, J. L., O'Neill, R. J., Timp, W., Zook, J. M., Schatz, M. C., Eichler, E. E., Miga, K. H., and Phillippy, A. M. The complete sequence of a human genome. *bioRxiv* (2021).
